# Platinum(iv) dihydroxido diazido *N*-(heterocyclic)imine complexes are potently photocytotoxic when irradiated with visible light†
†Electronic supplementary information (ESI) available: Materials and methods, synthesis and characterisation data, X-ray crystallographic tables, biological data. CCDC deposits 1904755 (**28**), 1904751 (**20**), 1904753 (**26**), 1904752 (**23**), 1904754 (**32**) contain the crystallographic data. For ESI and crystallographic data in CIF or other electronic format see DOI: 10.1039/c9sc02644d


**DOI:** 10.1039/c9sc02644d

**Published:** 2019-08-08

**Authors:** Evyenia Shaili, Luca Salassa, Julie A. Woods, Guy Clarkson, Peter J. Sadler, Nicola J. Farrer

**Affiliations:** a Chemistry Research Laboratory , University of Oxford , 12 Mansfield Road , Oxford , OX1 3TA , UK . Email: Nicola.Farrer@chem.ox.ac.uk ; Email: p.j.sadler@warwick.ac.uk ; Tel: +44 (0)1865 285131; b Department of Chemistry , University of Warwick , Gibbet Hill Road , Coventry , CV4 7AL , UK; c Photobiology Unit , Department of Dermatology and Photobiology , Ninewells Hospital , Dundee , DD1 9SY , UK

## Abstract

A series of *trans*-di-(*N*-heterocyclic)imine dihydroxido diazido Pt^IV^ complexes of the form *trans*,*trans*,*trans*-[Pt(N_3_)_2_(OH)_2_(L_1_)(L_2_)] where L = pyridine, 2-picoline, 3-picoline, 4-picoline, thiazole and 1-methylimidazole have been synthesised and characterised, and their photochemical and photobiological activity evaluated. Notably, complexes **19** (L_1_ = py, L_2_ = 3-pic) and **26** (L_1_ = L_2_ = 4-pic) were potently phototoxic following irradiation with visible light (420 nm), with IC_50_ values of 4.0 μM and 2.1 μM respectively (A2780 cancer cell line), demonstrating greater potency than the previously reported complex **1** (L_1_ = L_2_ = py; 6.7 μM); whilst also being minimally toxic in the absence of irradiation. Complexes with mixed *N*-(heterocyclic)imine ligands **19** and **20** (L_1_ = py, L_2_ = 4-pic) were particularly photocytotoxic towards cisplatin resistant (A2780cis) cell lines. Complex **18** (L_1_ = py, L_2_ = 2-pic) was comparatively less photocytotoxic (IC_50_ value 14.5 μM) than the other complexes, despite demonstrating the greatest absorbance at the irradiation wavelength and the fastest half-life for loss of the N_3_ → Pt LMCT transition upon irradiation (*λ*_irr_ = 463 nm) in aqueous solution. Complex **29** (X_1_ = X_2_ = thiazole) although potently phototoxic (2.4 μM), was also toxic towards cells in the absence of irradiation.

## Abstract 2

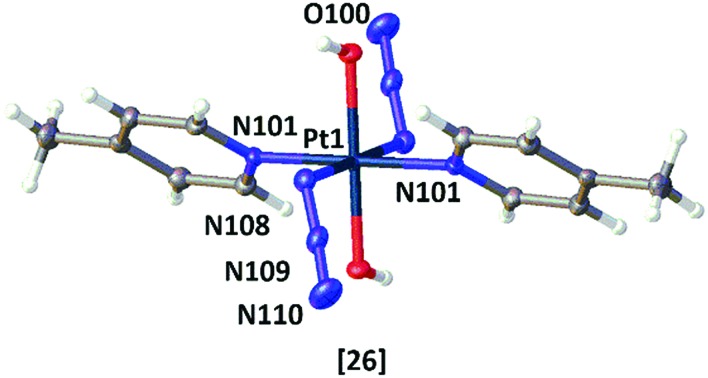
 *Trans*,*trans*,*trans*-[Pt(N_3_)_2_(OH)_2_(4-picoline)_2_] is potently photocytotoxic (*λ*_irr_ = 420 nm) towards cancer cell lines whilst being minimally toxic in the absence of irradiation.

## Introduction

**Fig. 1:**
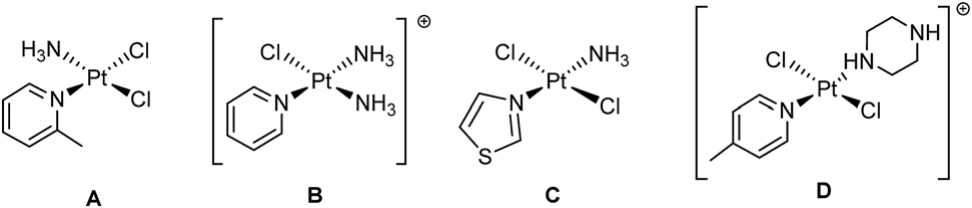
Pt(ii) compounds (A) picoplatin, (B) pyriplatin, (C) *trans*-[PtCl_2_(NH_3_)(thiazole)] and (D) *trans*-[PtCl_2_(4-pic)(piperazine·H)]^+^ which exhibit promising anti-cancer properties.

For cancers treated with chemotherapy, approximately half of patients currently receive a platinum(ii) drug, typically in combination with other therapies.^1^,^2^ The widely-used platinum(ii) complexes cisplatin,^3^ carboplatin^4^ and oxaliplatin^5^,^6^ all include ammine or aliphatic amine ligands, and exhibit potent activity against a number of different cancers. Development of resistance to treatment is a serious problem for platinum-based drugs, and a number of strategies have been explored to combat this, including the use of alternative ligand classes and geometries.^7^ In addition to the use of aliphatic amines, Pt(ii) complexes incorporating *N*-heterocyclic amines also show promise: picoplatin (AMD 473, A) which incorporates 2-picoline has been studied in clinical trials for the treatment of small cell lung cancer in 2007 (Fig. 1).^8^

Picoplatin was developed to tackle the problem of platinum drug deactivation by S-donor molecules, such as glutathione, which can lead to the development of resistance. The steric hindrance caused by the methyl substituent on the pyridyl ring in close proximity to the Pt centre is thought to reduce the rates of side-reactions which can diminish drug potency.^9^ The pyridyl-complex pyriplatin (**B**) is highly cytotoxic towards a number of tumour cell lines; forming monofunctional Pt-DNA adducts which are proposed to evade DNA repair and inhibit transcription. The positive charge on the complex makes cellular uptake through organic cation transporters more likely than through passive diffusion or copper transporters – uptake mechanisms which are implicated for cisplatin – giving it the potential to be effective against cisplatin-resistant lines.^10^ Studies in zebrafish suggest that monofunctional complexes such as pyriplatin and phenanthriplatin may also generate less severe auditory side-effects (ototoxicity), compared with cisplatin.^11^ In addition to the ligands themselves, the use of aromatic pyridine-like ligands or substituted amines in a *trans* rather than a *cis* geometry in a Pt complex can give rise to cytotoxic activity in cisplatin-resistant cell lines.^12^,^13^

*Trans* complexes such as *trans*-[PtCl_2_(NH_3_)(thiazole)] (Fig. 1C) which include non-pyridyl heterocyclic amines such thiazole also demonstrate a good combination of cytotoxicity, aqueous solubility and *in vivo* activity,^14^ and *trans*-[PtCl_2_(4-pic)(piperazine·H)]^+^ (Fig. 1D) which incorporates picolyl and piperazine ligands is also highly cytotoxic towards cancer cells, with a mechanism of action distinct from cisplatin.^15^

Another strategy in the development of more selective anti-cancer drugs is the use of Pt^IV^ prodrugs;^16^ oxidation of square-planar Pt^II^ complexes to octahedral Pt^IV^ complexes incorporates two additional ligands into the axial positions. These low-spin 5d^6^ Pt^IV^ complexes are kinetically more inert than their Pt^II^ precursors, minimising unwanted side-reactions in advance of reduction *in cellulo*.^17^–^20^

Pt^IV^ azido complexes are a promising class of stimuli-responsive prodrugs which may be non-toxic in the dark but exhibit potently photocytoxic effects when irradiated with visible light.^21^ Complexes with amine ligands in a *trans* geometry can be more photocytotoxic than their corresponding *cis* isomers,^22^ and replacing aliphatic amines (*e.g*. NH_3_) with pyridyl ligands can enhance the photocytotoxic activity of a Pt^IV^ diazido complex when irradiated with longer – and therefore more clinically relevant – wavelengths of light; *trans*,*trans*,*trans*-[Pt(py)(NH_3_)(N_3_)_2_(OH)_2_] and *trans*,*trans*,*trans*-[Pt(py)_2_(N_3_)_2_(OH)_2_]^23^ exhibit IC_50_ values of 25.4 μM and 6.7 μM, respectively, (A2780 ovarian cancer cell line) when irradiated with visible light (420 nm) irradiation, whilst being non-toxic in the absence of irradiation. Use of other *N*-heterocyclic amine ligands like thiazole in *e.g. trans*,*trans*,*trans*-[Pt(N_3_)_2_(OH)_2_(methylamine)(thiazole)] can also result in potently photocytotoxic complexes.^24^

**Fig. 2:**
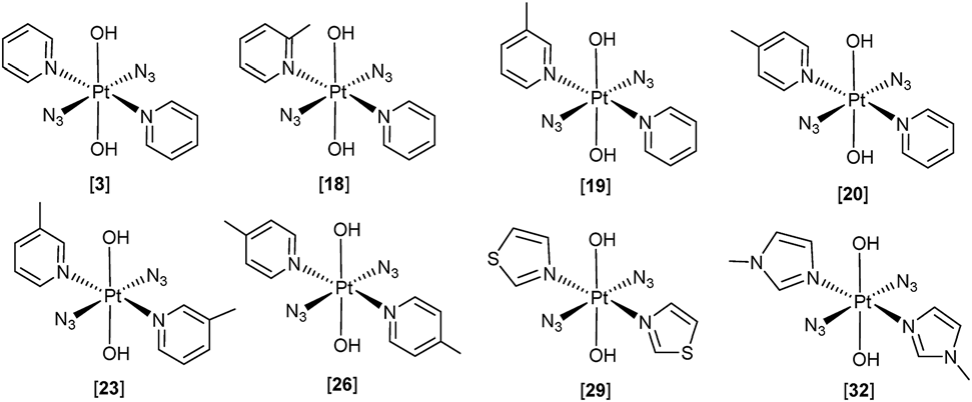
Pt^IV^ diazido complexes reported previously (**3**), and studied here: *trans*,*trans*,*trans*-[Pt(N_3_)_2_(OH)_2_(2-picoline)(pyridine)] (**18**), *trans*,*trans*,*trans*-[Pt(N_3_)_2_(OH)_2_(3-picoline)(pyridine)] (**19**), *trans*,*trans*,*trans*-[Pt(N_3_)_2_(OH)_2_(4-picoline)(pyridine)] (**20**), *trans*,*trans*,*trans*-[Pt(N_3_)_2_(OH)_2_(3-picoline)_2_] (**23**), *trans*,*trans*,*trans*-[Pt(N_3_)_2_(OH)_2_(4-picoline)_2_] (**26**), *trans*,*trans*,*trans*-[Pt(N_3_)_2_(OH)_2_(thiazole)_2_] (**29**), *trans*,*trans*,*trans*-[Pt(N_3_)_2_(OH)_2_(1-methylimidazole)_2_] (**32**).

These structure–activity relationships suggested that Pt^IV^ azido complexes with *N*-heterocyclic amine ligands in a *trans* geometry require further investigation as potential photactivatable anti-cancer complexes. Here we present the synthesis, characterisation, photochemical and photobiological evaluation of a series of novel *trans*-di-(*N*-heterocyclic)imine dihydroxido diazido Pt^IV^ complexes (Fig. 2).

## Results

### Synthesis and characterisation

The precursor Pt^II^ complexes **1** and **2**,^23^**8–11**, **21**, **24**, **27** and **30** were synthesised using established methods (see ESI†).^25^ Complexes **3**,^23^**8–11**, **21**, **24**,^26^ and complexes **27** and **30** (ref. 27) have been reported previously. The novel Pt^IV^-diazido complexes **18**, **19**, **20**, **23**, **26**, **29** and **32** were synthesised from their Pt^II^-diazido precursors *via* H_2_O_2_ oxidation, and characterised by ^1^H, ^13^C, ^195^Pt-NMR spectroscopy, ESI-MS and UV-vis spectroscopy (ESI).

### X-ray diffraction

#### N.B. Pt-azido atom labelling: Pt–N_α_–N_β_–N_γ_

**Fig. 3:**
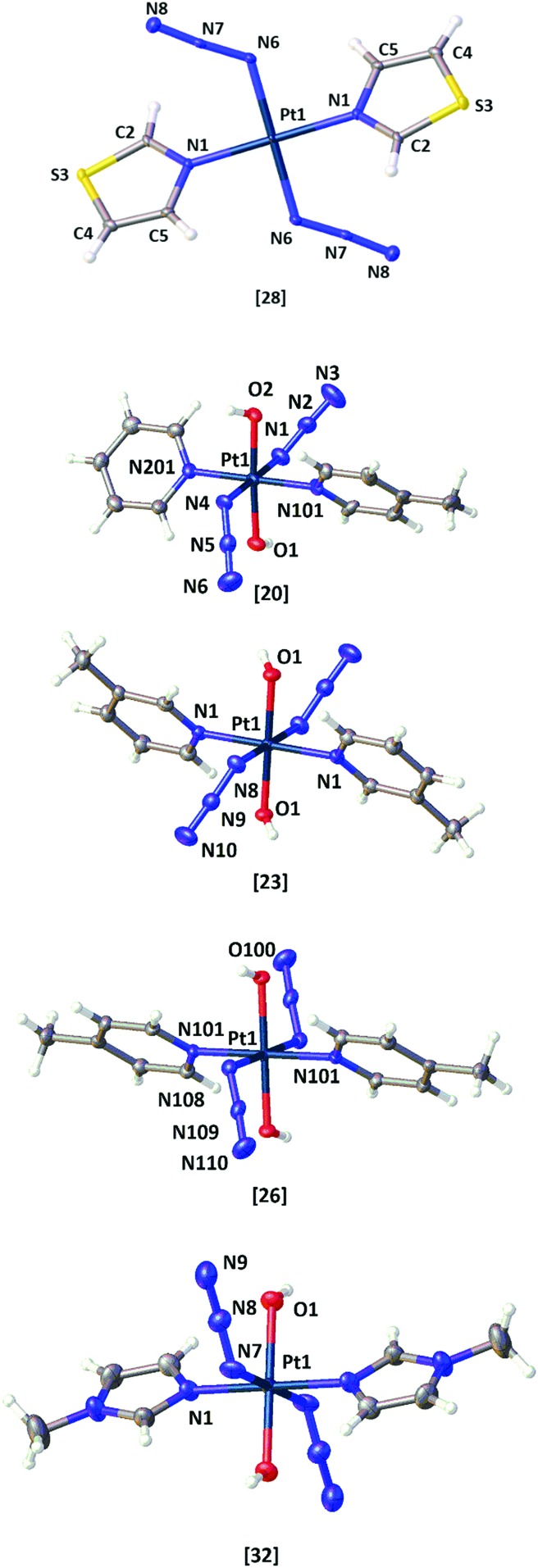
X-ray crystallographic structures of: *trans*-[Pt(N_3_)_2_(tz)_2_] **28**; *trans*,*trans*,*trans*-[Pt(N_3_)_2_(OH)_2_(py)(4-pic)] **20**; *trans*,*trans*,*trans*-[Pt(N_3_)_2_(OH)_2_(3-pic)_2_] **23**; *trans*,*trans*,*trans*-[Pt(N_3_)_2_(OH)_2_(4-pic)_2_] **26**; *trans*,*trans*,*trans*-[Pt(N_3_)_2_(OH)_2_(mim)_2_] **32**. Thermal ellipsoids are displayed at 50% probability; figures were generated using Olex2.^28^

Single crystals suitable for X-ray diffraction studies were obtained for the novel Pt^II^ complex *trans*-[Pt(N_3_)_2_(tz)_2_] **28** (where tz = thiazole), and confirmed that the thiazole ligand is *N*- rather than *S*-coordinated to the Pt^II^ centre (Fig. 3 and Tables S1–S3†). X-ray crystallographic structures were also obtained for four of the novel Pt^IV^ azido picoline and imidazole complexes: **20** (py, 4-pic); **23** (3-pic, 3-pic); **26** (4-pic, 4-pic) and **32** (mim, mim), Fig. 3; crystals suitable for X-ray diffraction were grown as detailed in the ESI.† Crystallographic data for **20**, **26**, **23** and **32** are summarized in Tables S4–S8.† The crystal structures of the Pt^IV^ complexes all have an octahedral geometry with an [N_4_O_2_] coordination sphere around the metal centre. The structures of complexes **23** (3-pic, 3-pic), **26** (4-pic, 4-pic) and **32** (mim, mim) which each contain two identical amine ligands are highly symmetrical, with an O–Pt–O bond angle of 180.0(0)°. In contrast, the structure of complex **20** (py, 4-pic) is slightly distorted, with an O–Pt–O angle of 176.5(2)°. The two symmetrical picolyl complexes **23** and **26** have similar bond lengths and angles; with lengths of Pt–N_α_ (2.048(2) Å; 2.046(2) Å), N_α_–N_β_ (1.217(3) Å; 1.225(4) Å) and N_β_–N_γ_ (1.147(3) Å; 1.152(4) Å) and angles N_α_–N_β_–N_γ_ (174.5(2)°; 175.6(3)°) and Pt–N_α_–N_β_ (115.56(16)°; 115.3(2)°) respectively. Complex **20** (py, 4-pic) showed similar Pt–N_α_ bond lengths to **23** and **26** (2.021(8) Å/2.038(7) Å) whereas in complex **32** (mim, mim) this bond was slightly elongated to 2.051(5) Å. In both complex **32** and complex **20** the Pt–N_α_N_β_ angle was less acute (118.8(4)°; 118.3(6)°/117.2(6)° respectively) when compared with the symmetric *bis* picolyl complexes.

### Solution chemistry

#### Solubility

The *trans*-di-(*N*-heterocyclic)imine dihydroxido diazido Pt^IV^ complexes showed considerable variation in their maximum aqueous solubilities (Table 1), and were stable in D_2_O in the dark at ambient temperature (20–25 °C) for >2 weeks, as determined by ^1^H-NMR spectroscopy.

**Table 1:** Maximum aqueous solubility of the Pt^IV^ diazido complexes at 20 °C

Complex	Solubility/mM
**3**	34
**18**	20
**19**	20
**20**	20
**23**	3
**26**	4
**29**	1
**32**	82

#### ESI-MS

**Fig. 4:**
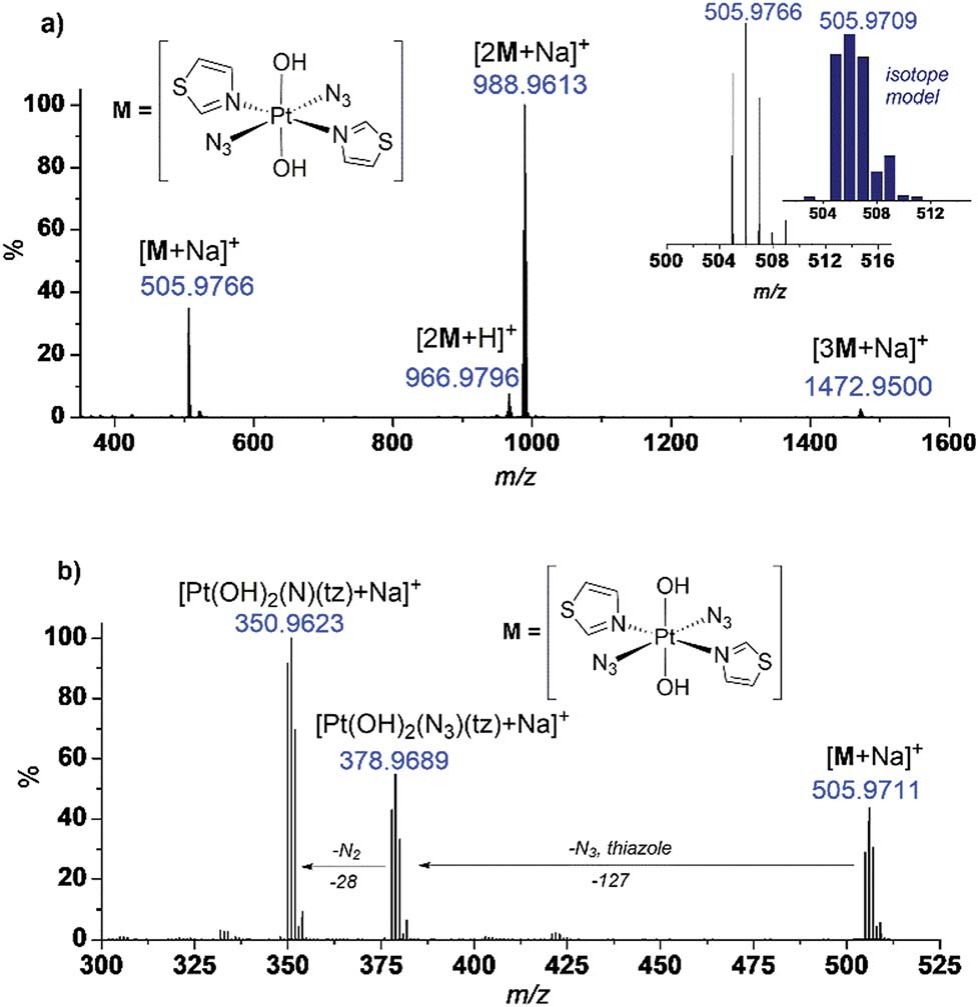
(a) ESI-MS of complex *trans*,*trans*,*trans*-[Pt(OH)_2_(N_3_)_2_(tz)_2_] (**29**) in H_2_O; (b) MS/MS of species [**29** + Na]^+^ (505.9711 *m*/*z*) revealing loss of neutral fragments N_3_˙, N_2_ and the thiazole ligand.

As commonly observed for Pt^IV^ diazido complexes, the complexes were detected by ESI-MS as a series of sodiated adducts, as exemplified by the thiazole complex *trans*,*trans*,*trans*-[Pt(OH)_2_(N_3_)_2_(tz)_2_] (**29**) (Fig. 4a). Speciation typically varies between H^+^ and Na^+^ adducts, depending on the mass spectrometer.

#### MSMS studies

Collision-induced dissociation can provide insight into the stability of a metal complex and the lability of the various ligands attached to the metal centre. Selective MSMS fragmentation of the sodiated complex [**29** + Na]^+^ (Fig. 4b) gave similar fragmentation products to those which we previously reported for the dipyridyl complex, *trans*,*trans*,*trans*-[Pt(OH)_2_(N_3_)_2_(py)_2_] (**3**);^23^ in both cases fragmentation products were detected, *e.g.* for complex **29:** [Pt(OH)_2_(N_3_)(tz) + Na]^+^ (378.97 *m*/*z*) following loss of the aromatic ligand and N_3_˙, and [Pt(OH)_2_(N)(tz) + Na]^+^ (350.96 *m*/*z*) following further loss of N_2_. The necessity of the products to be charged in order to be detected by ESI-MS does mean that these species represent only the positively-charged subset of the fragmentation products which are likely to be formed.

#### NMR spectroscopy

The complexes were characterised by ^1^H, ^13^C and ^195^Pt NMR spectroscopy. ^195^Pt NMR resonances for the novel Pt^IV^ complexes were all observed within the range 954–987 ppm (D_2_O), apart from complex **18** (py, 2-pic), for which the resonance was significantly more deshielded, being observed at 1132 ppm.

#### UV-vis absorption spectra

**Fig. 5:**
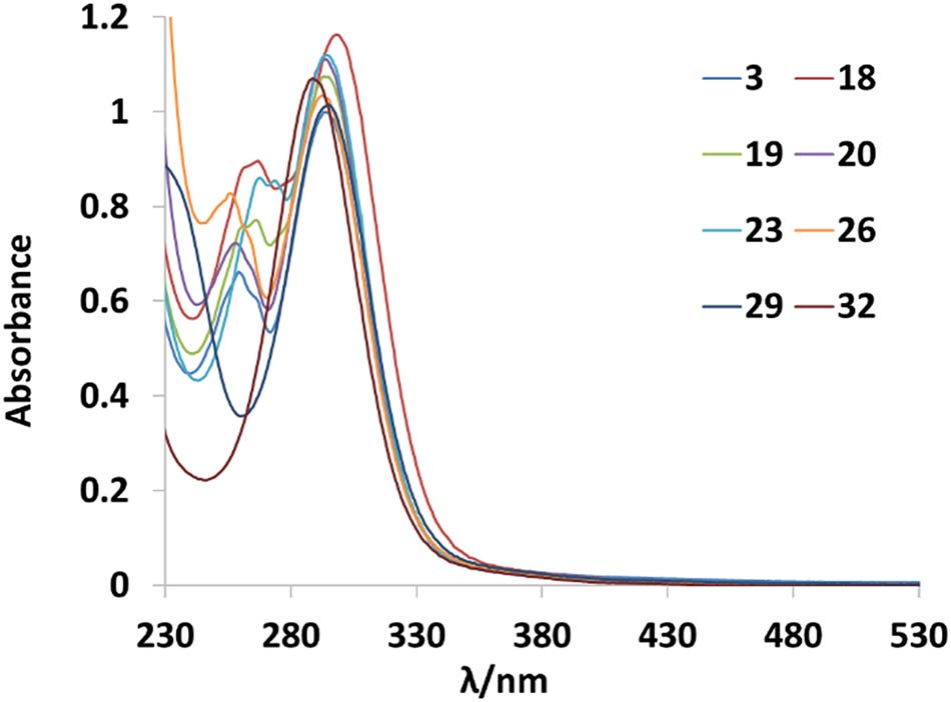
UV-vis spectra of eight Pt(iv)-diazido complexes (60 μM, H_2_O).

The UV-vis spectra of the Pt^IV^ diazido complexes in H_2_O are overlaid in Fig. 5. The absorption maxima (*λ*_max_) corresponding to the LMCT N_3_ → Pt transition for the complexes lies in the range 290–297 nm. Complex **18** (py, 2-pic) has the longest wavelength absorption maximum (297 nm), whereas **32** (mim, mim) has the shortest (290 nm). Complexes incorporating pyridyl derivatives have two main absorption bands in the UV region, whereas those incorporating thiazole or 1-methylimidazole have only one main absorbance band.

#### TD-DFT

**Fig. 6:**
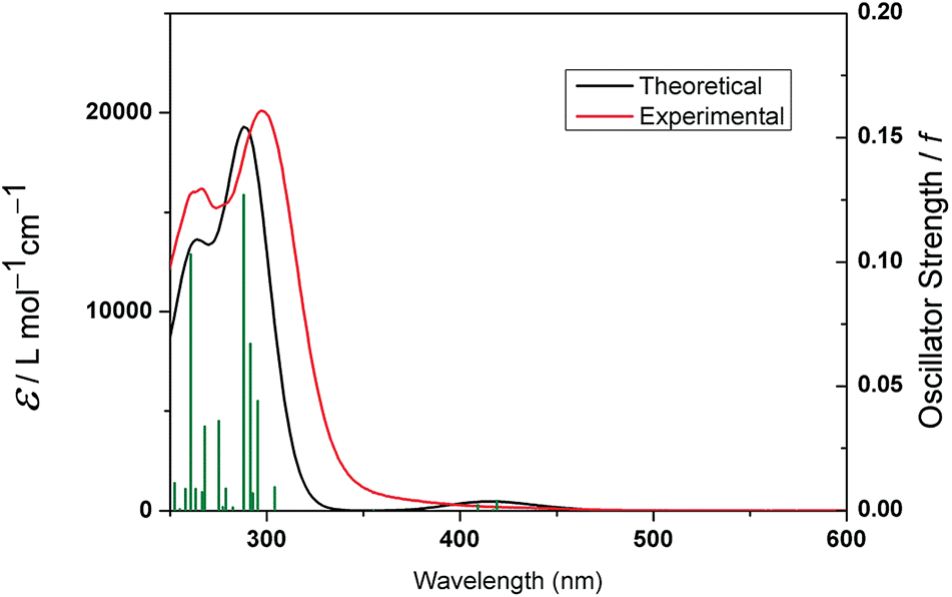
Calculated (red) and experimental (black) absorption spectra of **18**. The excited states are shown as vertical green bars with heights equal to the extinction coefficients. The theoretical spectrum was obtained using GAUSSUM 2.2.^29^

Because complex **18** (py, 2-pic) had the longest *λ*_max_, DFT (restricted and unrestricted), TDDFT calculations were carried out to characterize the singlet and triplet excited states. The theoretical UV-vis spectrum was simulated by the calculation of 32 singlet states using water as a solvent. The theoretical and experimental UV-vis spectra show reasonable agreement, with the absorption maximum underestimated by 9 nm (experimental: 297 nm, 20 091 M^–1^ cm^–1^, theoretical 288 nm, 19 282 M^–1^ cm^–1^) and the shoulder with only 1 nm difference (theoretical: 267 nm, 13 520 M^–1^ cm^–1^; experimental: 268 nm, 16 061 M^–1^ cm^–1^) (Fig. 6). As was previously reported for complex **3**, these transitions can be assigned to dissociative ^1^LMCT (N_3_ → Pt) and mixed ^1^LMCT/^1^IL (OH → Pt, N; IL = interligand) transitions. The singlet excited states as well as the percentage contribution are presented in Tables S9, S10 and Fig. S1.†

### Photochemistry

#### Solution photochemistry

**Fig. 7:**
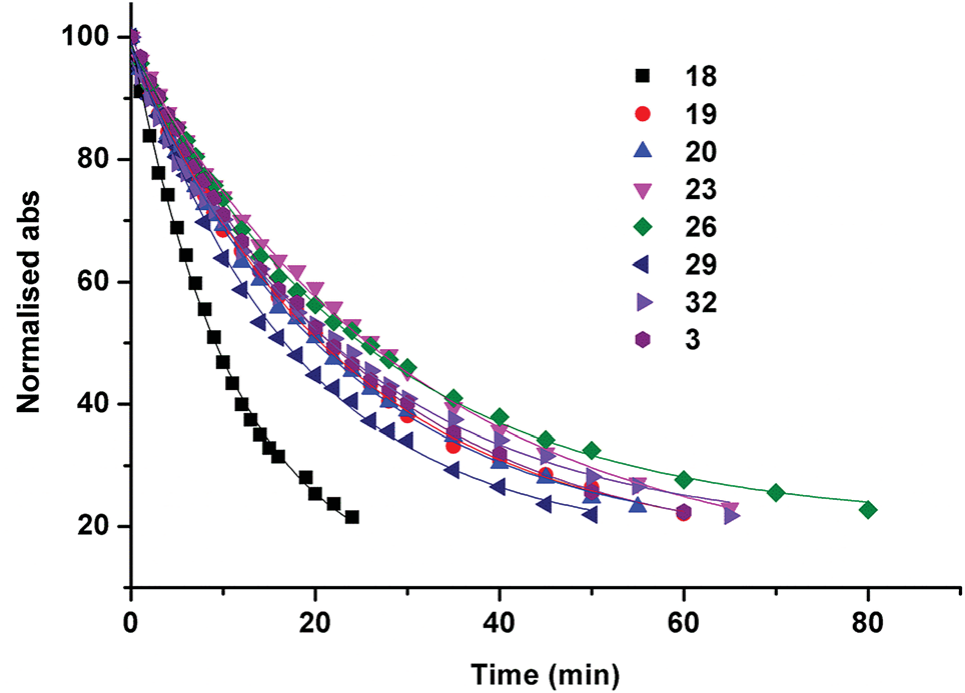
Rate of decrease of the normalised absorbance (290–297 nm) for the Pt(iv) diazido complexes corresponding to the loss of the N_3_ → Pt LMCT band to an extent of *ca.* 22% of the original intensity, when irradiated with blue light (463 nm, 57 mW cm^–2^).

Solutions of the Pt^IV^ diazido complexes (60 μM, H_2_O) were irradiated with blue light (463 nm, 57 mW cm^–2^). The decrease in intensity of the *λ*_max_ N_3_ → Pt band was monitored over time for each complex by UV-vis spectroscopy until *ca.* 22% of the original intensity remained (Fig. S2†). The data were fitted to *y* = *y*_0_ + Ae^Rox^, with *r*^2^ > 0.98 (Fig. 7). The fastest half-life of photodecomposition was observed for complex **18** (py, 2-pic); 2.5-fold faster than for the slowest for complexes **23** (3-pic, 3-pic) and **26** (4-pic, 4-pic) (Table S11†). The photosensitivity of complexes **18**, **19** and **20** was also investigated with longer-wavelength green light (*λ* = 517 nm, 30 mW cm^–2^); despite minimal visible absorption at this wavelength the complexes clearly demonstrated photoinduced loss of the N_3_ → Pt band, although the process was significantly slower (>6 hours) to achieve a conversion comparable to that observed with 463 nm irradiation (Fig. S3†).

Irradiation of complexes **19**, **20**, **23**, **26** and **32** (2 mM in PBS/D_2_O pH* 7.4, 1 mM for **29**, due to low solubility) with 420 nm light (1 h, 24.7 mW cm^–2^) in the presence of 5′-GMP (2 mol eq.) resulted in formation of 5′-GMP platinum adducts (determined by ^1^H NMR spectroscopy). Photoejection of the *N*-heterocyclic ligand was not observed for any complex. The percentage of bound 5′-GMP was quantified by integration of the diagnostic 5′-GMP sugar C_1_ protons in the ^1^H NMR spectral region 5.50–6.00 ppm (Table S12†). The highest conversion to new 5′-GMP adducts was seen for **29** (tz, tz) (80%), and the lowest for **32** (mim, mim) (48%) with the remaining compounds showing intermediate conversions. Complex **18** showed moderate 5′-GMP binding (52%).

Complex **18**, as the most rapidly photoreduced complex in the absence of 5′-GMP (Fig. 7), was irradiated in the presence of 5′-GMP (9 mM in PBS/D_2_O pH* 7.4, 2 mol eq. 5′-GMP) with 420 nm light (45 min, 7 mW cm^–1^) and was more extensively investigated by ^1^H, ^195^Pt NMR spectroscopy and LC-MS. The intensity of the ^195^Pt NMR spectroscopic signal corresponding to the Pt^IV^ starting material (1132 ppm) rapidly decreased (Fig. S4A†), and a new resonance corresponding to [Pt^II^(N_3_)(py)(2-pic)(5′-GMP)] appeared at –2195 ppm. A smaller signal (–2110 ppm) was also detected upfield of the main resonance in the Pt^II^ region (Fig. S4B†). After two weeks in the dark, the signal at –2110 ppm had disappeared, and a new broad resonance corresponding to the *bis* 5′-GMP adduct [Pt^II^(py)(2-pic)(5′-GMP)_2_] appeared at –2280 ppm (Fig. S4C†). The samples were analysed by LC-MS shortly after irradiation, and again after two weeks, confirming the proposed speciation and the relative increase in concentration of the *bis* adduct (Fig. S5†). This is in contrast to our observations for **3**, which rapidly formed two approximately equal intensity signals corresponding to the *mono* (–2212 ppm) and *bis* (–2288 ppm) substituted 5′-GMP adducts.^23^

### Phototoxicity

The novel *trans*-di-(*N*-heterocyclic)imine dihydroxido diazido Pt^IV^ complexes Pt^IV^ complexes were evaluated against human ovarian (A2780), cisplatin-resistant ovarian (A2780cis) and oesophageal (OE19) cancer cell lines, and the previously reported compounds (**FM165** and **3**) were also evaluated for comparison. Phototoxicity (IC_50_ values) and phototoxicity indices (PI) are shown in Table 2. Complexes **19**, **20**, **23**, **26**, and **32** were non-toxic in the absence of irradiation (defined as IC_50_ > 200 μM). Complex **18** (py, 2-pic) exhibited moderate dark toxicity in both of the cell lines in which it was evaluated (A2780 and A2780cis), and complex **29** (tz, tz) exhibited some dark toxicity in the A2780 cell line, but no toxicity in either of the A2780cis or OE19 lines.

**Table 2:** Phototoxicity of the Pt^IV^ complexes *trans*,*trans*,*trans*-[Pt(N_3_)_2_(OH)_2_(L_1_)(L_2_)] following irradiation with blue light (420 nm, 5 J cm^–2^, TL03, filtered <400 nm).Values are in μM

Complex	L_1_	L_2_	A2780			A2780cis			OE19		
			IC_50_	95% CI	^*a*^PI	IC_50_	95% CI	PI	IC_50_	95% CI	PI
**18**	py	2-pic	14.5	12.7–16.7	7.3^*d*^	15.5	11.2–21.5	5.3^*d*^	ND	ND	ND
**19**	py	3-pic	4.0	3.0–5.4	>51.5	3.3	1.9–5.9	>71.0	5.5	3.2–9.4	>38
**20**	py	4-pic	5.4	4.3–6.8	>38	4.6	3.8–5.6	>45	12.3	7.9–19.1	>17
**23**	3-pic	3-pic	7.2	6.0–8.7	>27.8	10.4	8.7–12.2	>19.3	ND	ND	ND
**26**	4-pic	4-pic	2.1	1.6–2.7	>95	4.1	3.3–5.1	>48.8	8.2	5.5–12.0	>24
**29**	Thiazole	Thiazole	2.4	2.2–2.7	48^*d*^	2.9	1.6–5.2	>71.0	7.6	5.4–10.7	>27
**32**	mim	mim	56.3	40.2–78.9	>3.7	164.2	Wide	>1.3	ND	ND	ND
**FM165** ^*c*^	py	NH_3_	25.4	22.2–29.1	>9.6	^*b*^ND	ND	ND	ND	ND	ND
**3**	py	py	6.7	3.6–13.7	>45	ND	ND	ND	8.4	ND	>45

^*a*^PI = phototoxic index (ratio of cytotoxicity following irradiation *vs.* in the dark). Explicit cytotoxicity values in the dark will be added to the ESI if they become available.

^*b*^ND = not determined.

^*c*^FM165 = *trans*,*trans*,*trans*-[Pt(N_3_)_2_(OH)_2_(NH_3_)(py)].

^*d*^= cytotoxic in the absence of irradiation. mim = 1-methylimidazole.

Aside from complex **18** (py, 2-pic); the py/*n*-pic family of complexes (**19**, **20**, **23**, **26**) all exhibited potent phototoxicity with minimal toxicity in the dark. Compared with **3** (py, py) as a benchmark,^23^**26** (4-pic, 4-pic) demonstrated greater phototoxicity in the lines tested, being 3-fold more potent towards the A2780 line (2.1 μM *vs.* 6.7 μM) with similar phototoxicity (8.2 μM (**26**) *vs.* 8.4 μM (**3**)) in the OE19 line. In the lines tested, complex **23** (3-pic, 3-pic) was less potent than **26** (4-pic, 4-pic), and within error, was similar to complex **3**.

The mixed ligand complexes **19** (py, 3-pic) and **20** (py, 4-pic) showed slightly greater phototoxicity than **3** (py, py) in the A2780 ovarian line (4.0 μM and 5.4 μM respectively compared to 6.7 μM (**3**)), but encouragingly, demonstrated greater photototoxicity in the cisplatin resistant line A2780cis (3.3 μM (**19**) and 4.6 μM (**20**)) than in the A2780 line. We previously determined that – with UVA irradiation – **3** was significantly less phototoxic in A2780cis (14.5 μM) than in A2780 (1.4 μM). Furthermore, **19** (py, 3-pic) showed the most potent phototoxicity (5.5 μM) towards OE19 cells of all the compounds tested.

Complex **29** (tz, tz) demonstrated potent phototoxicity (2.9 μM in A2780cis cells; 7.6 μM in OE19) compared with our previously reported thiazole complex *trans*,*trans*,*trans*-[Pt(N_3_)_2_(OH)_2_(NH_2_CH_3_)(thiazole)] (6.4 μM in A2780cis; 19.3 μM in OE19)^24^ with blue light (420 nm) irradiation, without any dark toxicity towards these cell lines. Complex **32** (mim, mim) demonstrated good aqueous solubility, but relatively low phototoxicity in the A2780 and A2780cis lines; due to this modest activity it was not evaluated against the OE19 cell line.

Despite potent photocytotoxicity under blue light irradiation (420 nm), complex **20** (py, 4-pic) showed no phototoxicity following irradiation with green light (515 nm) in either A2780 or OE19 cell lines.

## Discussion

### Synthesis and photochemistry

Seven novel *trans*-di-(*N*-heterocyclic)imine dihydroxido diazido Pt^IV^ complexes of form the *trans*,*trans*,*trans*-[Pt(N_3_)_2_(OH)_2_(L_1_)(L_2_)], where L_1_ and L_2_ = pyridine, 2, 3, 4-picoline, thiazole or 1-methylimidazole (**18**, **19**, **20**, **23**, **26**, **29** and **32**) have been synthesised from their Pt^II^-diazido precursors *via* H_2_O_2_ oxidation, and characterised by ^1^H, ^13^C, ^195^Pt-NMR spectroscopy, ESI-MS and UV-vis spectroscopy.

The single crystal X-ray crystal structures were obtained for one Pt^II^ diazido precursor (**28**) and four of the novel Pt(IV)-diazido complexes (**20**, **23**, **26**, **32**). Although slight distortions from octahedral geometry were observed in the complexes bearing two different *N*-heterocyclic amine ligands in *trans* position, the key bond lengths and angles within the structures are relatively similar to the previously reported *trans*-di-(*N*-heterocyclic)imine dihydroxido diazido Pt^IV^ complex **3**. Although an X-ray crystal structure was not been obtained for the Pt^IV^ azido *bis*-thiazole derivative, it is likely that N-coordination of the thiazole is retained in **29** following oxidation.

The Pt^IV^ azido compounds demonstrated significant variation in aqueous solubility over the range 1–82 mM depending on the *N*-heterocyclic amine ligands; complexes which were mixed-ligand isomers (py, *n*-pic) **18**, **19**, **20** exhibited similar aqueous solubility (20 mM) as did the *bis* (*n*-pic, *n*-pic) complexes (3–4 mM) and inclusion of two thiazole ligands significantly decreased the aqueous solubility (1 mM). These data indicate that the position of *n*-pic substitution has a negligible effect on the solubility.

UV-vis spectroscopy revealed that of all the complexes reported, **18** (2-pic, 2-pic) has the longest *λ*_max_ in the UV-vis absorption spectrum. Consistent with this, irradiation (463 nm) revealed **18** to be the most rapidly photoreduced complex, and DFT and TDDFT calculations showed higher intensity transitions for **18** in the 410–470 nm region compared to the previously reported complex **3** (py, py). Irradiation (420 nm) of the novel Pt^IV^ complexes in the presence of 5′-GMP resulted in the rapid formation of both *mono* and *bis* 5′-GMP Pt adducts, with the exception of the sterically crowded complex **18** which initially formed only the *mono* adduct [Pt(N_3_)(2-pic)(py)(5′-GMP)], whilst a smaller amount of *bis*-substituted adduct [Pt(2-pic)(py)(5′-GMP)_2_] formed significantly more slowly. This photochemical speciation was significantly different from that observed for **3** (py, py) which we previously demonstrated forms both *mono* and *bis* 5′-GMP adducts immediately.^23^ The reduction potential of *cis*,*trans*,*cis*-[Pt^IV^Cl_2_(OH)_2_(NH_3_)(L)] has been reported to increase by 56 mV by replacing L = pyridine with 2-picoline: attributed to the steric clash between the *N*-ligand and the platinum, destabilising the Pt–O bond and facilitating reduction to Pt(ii).^30^ We reasoned that such destabilization may be beneficial for the photochemical reduction of Pt(iv)–diazido complexes if sufficient stability in the dark could be retained.

Attempts to obtain crystals of **18** suitable for diffraction were unsuccessful. Nevertheless, the DFT-optimized geometry of **18** (Table S9†) resembled the asymmetries in the structure of **20**, albeit with slightly overestimated bond distances, particularly for Pt–O bonds.

### Phototoxicity

The choice of *N*-heterocyclic amine ligands plays a crucially important role in the photochemical and photobiological properties of *trans*-di-(*N*-heterocyclic)imine dihydroxido diazido Pt^IV^ complexes; variation in the position of a single methyl group within the series of *n*-pic complexes is sufficient to destabilise a complex (**18**: py, 2-pic) such that it becomes cytotoxic in the absence of irradiation towards both A2780 and A2780cis ovarian cancer cell lines. Variations in cytotoxicity between picoline analogues has precedent; for *trans*-[Pt^II^X_2_(3-pic)_2_] and *trans*-[Pt^II^X_2_(4-pic)_2_] (X = Cl^–^, OAc^–^) the 4-picoline complexes were more cytotoxic than 3-pic derivatives (Pam 212 and Pam 212-*ras* cell lines).^31^ In general, changing the position of a substituent on pyridine ligands has a significant effect on the cytotoxic properties; evaluation of *trans*-[PtCl_2_(3-acetylpyridine)_2_] and *trans*-[PtCl_2_(4-acetylpyridine)_2_] in a range of cell lines showed that the 4-acetylpyridine complex was more cytotoxic.^32^

Phototoxicity studies (*λ*_irr_ 420 nm) of the (py)(*n*-pic) series of complexes in different cell lines showed a general trend from most phototoxic to least phototoxic: **26** (4-pic, 4-pic) > **19** (py, 3-pic) > **20** (py, 4-pic) > **3** (py, py) > **23** (3-pic, 3-pic) > **18** (py, 2-pic). Despite already being cytotoxic in the absence of irradiation and the most rapidly photoreduced in solution, **18** (py, 2-pic) was the least phototoxic of this series; being nearly 7-fold less phototoxic than **26** (4-pic, 4-pic; A2780). Although the inclusion of the sterically demanding 2-picoline ligand results in a shift of LMCT band towards longer wavelengths and also in rapid photodecomposition of **18** (*λ*_irr_ 420 nm), this does not appear to translate to greater phototoxicity *in cellulo*. This may be due to excessive crowding close to the platinum centre and the slow formation of bifunctional adducts for **18** when compared with other complexes, as indicated by 5′-GMP binding studies. Picoplatin (Fig. 1) – which also contains 2-picoline – also forms DNA crosslinks and binds plasma proteins more slowly than cisplatin. However, picoplatin is likely to form different DNA lesions compared to **18** due to its *cis* geometry, and the NH_3_ of picoplatin is also less sterically demanding than the pyridine of **18**.

Encouragingly, little or no cross-resistance was observed for the mixed ligand complexes **19** (py, 3-pic) and **20** (py, 4-pic) in the cisplatin-resistant ovarian cancer cell line (A2780cis) than towards the ovarian (A2780) line. In contrast, the *bis* ligand complexes: **23** (3-pic, 3-pic) and **26** (4-pic, 4-pic) and **32** (mim, mim) and the previously reported **3** (py, py – with UVA light)^23^ all demonstrated relatively lower phototoxicity against the cisplatin resistant line (A2780cis) than the A2780 line. This indicates that the use of these mixed ligand systems may be effective at circumventing cisplatin-resistance; however, investigation of a wider panel of cell lines – particularly those which are more closely representative of patient-derived ovarian tumours than A2780 cells^33^ will clarify if this efficacy is more widely observed.

Although all the complexes were slightly less potent towards the oesophageal line than the ovarian cancer cell lines, the phototoxicity of **19** (py, 3-pic; 5.5 μM) towards the OE19 oesophageal cancer cell line is notable. Oesophageal cancer is one of the most deadly cancers worldwide because of its extremely aggressive nature and poor survival rate. Ranking sixth among all cancers in mortality, it is increasing in incidence in Western populations.^34^ It is also a superficial cancer which may benefit from photochemotherapy with light of shorter wavelengths (*e.g.* blue rather than green) to prevent damage to underlying healthy tissue, since light penetration is wavelength-dependent.^35^

Our previously reported thiazole complex (tz, methylamine) showed no dark cytotoxicity (IC_50_ > 232.9 μM) and good phototoxicity under 420 nm irradiation (IC_50_: 28.2 (A2780); (A2780cis) 6.4; (OE19) 19.3 μM).^24^ Although the *bis* thiazole complex **29** (tz, tz) reported here showed at least 2-fold greater phototoxicity in all three cell lines, **29** was also slightly cytotoxic in the absence of irradiation towards A2780 cells only (IC_50_ 115.2 μM; phototoxic index = 48). Pt^IV^ diazido complexes which are cytotoxic towards some cell lines in the absence of irradiation (*e.g.***18**, **29**) could potentially be derivatised and developed as redox-activatable Pt^IV^ prodrugs (*e.g.* satraplatin, which has been evaluated in Phase III trials for prostate cancer),^2^ particularly if they show suitable pharmacological characteristics (*e.g*. good solubility). However, the complexes would need to be potently (IC_50_ < 10 μM) cytotoxic which may be achievable through judicious axial ligand choice. Predicting the biological rates of reduction of Pt^IV^ complexes (*e.g.* by cytochrome c, metallothioneins, glutathione, ascorbate) is not straightforward;^36^ reduction potentials measured in solution do not necessarily correlate with rates of reduction by reducing agents.^37^ Reduction can occur through either inner or outer sphere mechanisms, depending on the bridging capabilities of ligands.^38^ If there are other ligands present which can bridge to reducing agents to facilitate reduction (*e.g.* Cl, OH) then complexes with axial halide (Cl/I) groups are generally reduced most rapidly, followed by *bis* COOR complexes, then mixed OH/COOR, with OH/OH complexes reduced most slowly.^39^ However, trends in reduction rates and cytotoxicity are highly complex-dependent: mixed OH/COOR compounds incorporating cisplatin and ethacrynic acid show more facile reduction (which translates to greater *in vivo* activity) than the corresponding *bis* COOR complexes, despite the presence of Cl ligands.^40^ In its current form, complex **29** is not sufficiently cytotoxic in the absence of irradiation to be developed as a redox-active Pt^IV^ prodrug and since it exhibited modest aqueous solubility (1 mM). Any (axial) modifications made to improve cytotoxicity would also need to improve solubility.

Replacing the pyridyl, picolyl or thiazole ligands with methylimidazole significantly decreased the phototoxicity of the complex (**32**). Investigation of the lipophilicity and cellular accumulation of this complex is anticipated to shed more light on the likely reason(s) for the lower phototoxicity.

## Experimental

For materials, methods and procedures see ESI.†

## Conclusions

We have reported the synthesis and characterisation of seven novel *trans*-di-(*N*-heterocyclic)imine dihydroxido diazido Pt^IV^ complexes which have been characterised by ^1^H, ^13^C, ^195^Pt-NMR spectroscopy, ESI-MS and UV-vis spectroscopy. Photochemical and photobiological experiments show that complexes **19** (py, 3-pic), **20** (py, 4-pic), **23** (3-pic, 3-pic), **26** (4-pic, 4-pic), and **32** (mim, mim) are non-toxic in the absence of irradiation in the cell lines investigated. The picolyl complexes **19**, **20** and **26** were more potently phototoxic in all cell lines than our previously reported complex **3** (py, py) when irradiated with blue light. Based on their good aqueous solubility (20 mM), dark stability and potent phototoxicity – particularly towards cisplatin resistant cell lines – complexes **19** (py, 3-pic) and **20** (py, 4-pic) in particular warrant further investigation as photoactivatable Pt^IV^ prodrugs. Complex **19** also shows good activity towards the oesophageal cancer line. Investigation of the cellular accumulation of these Pt^IV^ azido complexes is anticipated to shed further light on the observed biological activity;^41^ these experiments are currently underway.

## Conflicts of interest

There are no conflicts to declare.

## Supplementary Material

Supplementary information

Crystal structure data
