# Supplementary material for: Platinum(iv) dihydroxido diazido N-(heterocyclic)imine complexes are potently photocytotoxic when irradiated with visible light
Source: Chem Sci. 2019 Aug 8;10(37):8610–7. doi: 10.1039/c9sc02644d (PMC6844273; doi:10.1039/c9sc02644d)
Supplement: Supplementary file 1 [file SC-010-C9SC02644D-s001.pdf]

Electronic Supplementary Material (ESI) for Chemical Science.
This journal is © The Royal Society of Chemistry 2019

Platinum(IV) dihydroxido diazido N-(heterocyclic)imine complexes are potently photocytotoxic when irradiated with visible light

Evyenia Shaili, Luca Salassa, Julie A. Woods, Guy Clarkson, Peter J. Sadler and Nicola J. Farrer

Electronic Supporting Information

Contents

General Procedures 3

Syntheses 7

Synthesis of *cis*-N-heterocyclic starting materials 7

Cis-[Pt(I)$_2$(pyridine)$_2$] (8) 7

Cis-[Pt(Cl)$_2$(pyridine)$_2$]) (9) 7

Cis-[Pt(I)$_2$(2-picoline)$_2$] (10) 7

Cis-[Pt(Cl)$_2$(2-picoline)$_2$] (11) 7

Synthesis of mixed *trans*-N-heterocyclic starting materials 8

Trans-[Pt(Cl)$_2$(pyridine)(n-picoline)], where n = 2, 3 or 4 8

Trans-[Pt(Cl)$_2$(py)(2-pic)] (12) 8

Trans-[Pt(Cl)$_2$(py)(3-pic)] (13) 8

Trans-[Pt(Cl)$_2$(py)(4-pic)] (14) 8

General procedure for synthesis of *trans*-[Pt(N$_3$)$_2$(py)(n-pic)] complexes 9

Trans-[Pt(N$_3$)$_2$(py)(2-pic)] (15) 9

Trans-[Pt(N$_3$)$_2$(py)(3-pic)] (16) 9

Trans-[Pt(N$_3$)$_2$(py)(4-pic)] (17) 9

General procedure for synthesis of *trans, trans, trans*-[Pt(N$_3$)$_2$(OH)$_2$(py)(n-pic)] complexes 10

Trans, trans, trans-[Pt(N$_3$)$_2$(OH)$_2$(py)(2-pic)] (18) 10

Trans, trans, trans-[Pt(N$_3$)$_2$(OH)$_2$(py)(3-pic)] (19) 10

Trans, trans, trans-[Pt(N$_3$)$_2$(OH)$_2$(py)(4-pic)] (20) 10

Syntheses of *bis trans*-N-heterocyclic complexes 11

Trans-[Pt(Cl)$_2$(3-pic)$_2$] (21) 11

Trans-[Pt(N$_3$)$_2$(3-pic)$_2$] (22) 11

Trans, trans, trans-[Pt(N$_3$)$_2$(OH)$_2$(3-pic)$_2$] (23) 11

Trans-[Pt(Cl)$_2$(4-pic)$_2$] (24) 11

Trans-[Pt(N$_3$)$_2$(4-pic)$_2$] (25) 12

Trans-[Pt(N$_3$)$_2$(OH)$_2$(4-pic)$_2$] (26) 12

Trans-[Pt(Cl)$_2$(tz)$_2$] (27) 12

Trans-[Pt(N_3)$_2$(tz)$_2$] (28) 12

Trans, trans, trans-[Pt(N_3)$_2$(OH)$_2$(tz)$_2$] (29) 13

Trans-[Pt(Cl)$_2$(1-methylimidazole)$_2$] (30) 13

Trans-[Pt(N_3)$_2$(1-methylimidazole)$_2$] (31) 13

Trans-[Pt(OH)$_2$(N_3)$_2$(1-methylimidazole)$_2$] (32) 14

X-ray crystal data for complexes 28, 20, 23 26 and 32 15

TD-DFT calculations 25

Irradiation studies with blue light 27

Irradiation studies with green light 29

References 34

General Procedures

Materials

$K_2[PtCl_4]$ was purchased from Precious Metals Online. HPLC-grade solvents and Millipore filtered H_2O were used for the preparation and purification of compounds by HPLC. All other reagents and consumables were purchased from commercial suppliers and used as received. (IM) indicates use of a nylon syringe filter (pore size 0.2 µM). All manipulations were carried out under reduced lighting and solutions were prepared stored and handled with minimal exposure to light. Complex **3** *trans,trans,trans*-[Pt(N_3)$_2$(OH)$_2$(py)$_2$] was synthesised from $K_2[PtCl_4]$ via complexes *trans*-[Pt(Cl$_2$)(py)$_2$] (**1**) and *trans*-[Pt(N_3)$_2$(py)$_2$] (**2**) successively, as previously reported.[1]

Methods

NMR spectroscopy. Due to the potential photosensitivity of the compounds, amberised NMR spectroscopy tubes (Goss Scientific) were used. ^{1}H, ^{13}C and ^{195}Pt NMR spectra were acquired at 298 K on Bruker AV-400 (1H: 399.10 MHz), Bruker DPX-400 (1H: 400.03 MHz), Bruker AVIII-600 (1H: 600.13 MHz) or Bruker Avance 700 spectrometers. Spectra were processed using Topspin 3.2. *J* values are quoted in Hz. **^{13}C NMR and ^{1}H NMR** were referenced internally to residual solvent or added 1,4-dioxane in the case of D_2O.[2] All chemical shift (δ) values are given in parts per million (ppm). **^{195}Pt NMR:** chemical shifts were externally referenced to K_2PtCl_6 in 1.5 mM HCl in D_2O (δ 0 ppm): for spectra of Pt^{IV} species directly bonded to quadrupolar ^{14}N, typical parameters used were d1 = 0 s, TD 2k, DE 10 µs, 256k scans. Data were processed with a LB of 50 Hz. **^{14}N NMR:** chemical shifts were externally referenced to [^{14}N]NH_4Cl (1.5 M) in 1 M HCl with a D_2O coaxial insert and processed with a qfil baseline correction.

Mass Spectrometry: Spectra were acquired on a Bruker Esquire 2000 Trap Spectrometer or Agilent 6130 single Quad, using an automatic sample delivery system. Data were processed using Data Analysis version 3.3 (Bruker Daltonics). **MS/MS experiments**: positive mode electrospray mass spectra were recorded using a MAXIS UHRQq- TOF (Bruker). **ICP-MS** was carried out on an Agilent 7500 Series spectrometer. Data acquisition was carried out on ICP-MS Top (version B.03.05) and analysis on Offline Data Analysis (version B.03.05). The standards were prepared from a stock of 1000 ppm Pt solutions

obtained from Sigma Aldrich, in 5% HNO_3 with miliQ water at the following concentrations: 200, 50, 10, 5, 1, 0.5, 0.2, 0.05, 0.01 ppb.

UV-visible absorption spectra: UV-vis spectra were recorded on a Perkin-Elmer Lambda 20 UV-visible spectrophotometer or a Varian Cary 300 UV-Vis spectrophotometer, in 1 cm path-length quartz cuvettes purchased from Starna Scientific.

Extinction coefficient determination: Extinction coefficients were determined using the Beer-Lambert law (A = εcl, where ε is the extinction coefficient ($M^{-1}cm^{-1}$), c the molar concentration and l the path length in cm). Pt concentrations for determination of extinction coefficients were measured by ICP-MS. For the shorter wavelengths, solutions of *ca*. 70 and 50 µM were used whereas for the longer wavelengths the concentrations employed were *ca* 2 mM and 1 mM, except in the case of complex **29**, where a solution of 0.6 mM was used.

HPLC (LC-MS) for monitoring the photochemistry of complex **20** was carried out on a Dionex 3000RS UHPLC coupled with a Bruker MaXis Q-TOF mass spectrometer using a ZORBAX Eclipse Plus C18 (5 µm particle size, 150 × 4.6 mm) column, with 1 mL/min flow rate and 45 µl injection volume. The mobile phase was H_2O/MeOH with 0.1% TFA. The wavelength of detection was 254 nm. The mass spectrometer was operated in electrospray positive mode with a scan range 50 – 2,000 m/z. Source conditions are, end plate offset at -500 V; capillary at -4500 V; nebulizer gas (N_2) at 1.6 bar; dry gas (N_2) at 8 L/min; dry temperature 180 °C. Ion tranfer conditions were: ion funnel RF at 200 Vp/p; multiple RF at 200 Vp/p; quadruple low mass set at 55 m/z; collision energy at 5.0 ev; collision RF at 600 Vp/p; ion cooler RF at 50-350, Vp/p; transfer time set at 121 µs; pre-pulse storage time 1 µs. Calibration was carried out with sodium formate (10 mM) through a loop injection of 20 µL of standard solution at the beginning of each run. The spectra were processed with Bruker Daltonics Data Analysis.

DFT and TDDFT calculations: All calculations were performed with the Gaussian 03 (G03) program[3] employing the DFT method, with PBE1PBE functionals.[4] The LanL2DZ basis set[5] and effective core potential were used for the Pt atom and the 6-31G**+ basis set[6] was used for all other atoms. Geometry optimizations for complex **18** in the ground state (S_0) and lowest-lying triplet state (T_1) were performed in the gas phase and the nature of all stationary points was confirmed by normal mode analysis. For the T_1 geometries the UKS method with the unrestricted PBE1PBE functional was employed. Thirty-two singlet excited states with the corresponding oscillator strengths were determined with a Time-dependent Density Functional Theory (TD-DFT)[7,8] calculation.

X-ray crystallography experimental details: In all cases, a suitable crystal was selected and mounted on a glass fibre with Fomblin oil and placed on an Xcalibur Gemini diffractometer with a Ruby CCD area detector. Diffraction data were collected with Mo-Kα radiation (λ = 0.71073 Å). The crystal was held at 100(2) K: (**28**, (nf2); **20** (es2), **26** (es4)); or 150(2) K (**23** (es10), **32** (es9)). Using Olex2[9] the structure was solved with the ShelXT[10] structure solution program using Intrinsic Phasing and refined with the ShelXL[11] refinement package using Least Squares minimisation. The crystal structures have been deposited in the Cambridge Crystallographic Data Centre under the accession numbers CCDC: 1904755 (**28**); 1904751 (**20**), 1904753 (**26**), 1904752 (**23**), 1904754 (**32**) respectively.

Irradiation methods and devices: Photoactivation of Pt complexes were carried out at 293 K. The light sources used were a LZC-ICH2 photoreactor (Luzchem Research Inc.) equipped with a temperature

controller and 3 UVA lamps (Hitachi, λ_{max} = 361 nm) or 6 Luzchem LZC-420 lamps (λ_{max} = 420 nm) with no other sources of light filtration, or LED light sources (BASETech model no. SP-GU10 230V~50 Hz 1.3-2.1 W) with λ_{max} = 463 nm or 515 nm.

Cell Studies

Maintenance. Human ovarian carcinoma (A2780), cisplatin-resistant ovarian carcinoma (A2780CIS) and oesophageal adenocarcinoma (OE19) cells were maintained in RPMI medium containing 10% Foetal Calf Serum (FCS). Cell lines were obtained from the European Collection of Animal Cell Cultures (ECACC). Cells were maintained in antibiotic-free medium in a humidified atmosphere of 95% air: 5% CO_2 and subcultured every 7-10 days. Mycoplasma checks were done using the Hoechst staining method. Cells were seeded into dishes the night before experiments at a density of 6 - 7×10^4 cells.cm^{-2}. Experiments were carried out in an adapted laboratory with ambient light levels kept below 1 lux (Solatell, UK).

Irradiation. Cells were irradiated by a bank of TL03 light sources (λ_{max} 420 nm), filtered to attenuate wavelengths below 400 nm. Irradiances, dosimetry and calibrations were traceable to the National Physical Laboratory.[12] Monochromatic light irradiation of the sample was performed using equipment consisting of a 1600W xenon arc light source and a grating monochromator. The apparatus was set to irradiate monochromatic light at 365, 400, 430, 500 and 530 nm. Filters were used to eliminate second order diffraction of shorter wavelengths from the specified longer wavelength monochromatic light.[13] Cells were irradiated in stirred suspension. Sham irradiated cells were treated identically to the test cells but were covered during irradiation.

Treatment. Test compounds were prepared in Earle's Balanced Salt Solution (EBSS) and filtered immediately before use. Photofrin was used as the positive control for visible light irradiation. The positive and negative controls were included on each plate in parallel with the complexes. Cell viability of untreated cells +/- visible light was >90%. Cells were treated for 1 h at 37ºC/5% CO_2 with EBSS containing the test compound, and then irradiated with 5 J.cm^{-2} visible radiation.

Phototoxic Index and Wavelength-Dependence. The phototoxic Index (PI) was determined 24 h after irradiation using the neutral red uptake assay;[14] following cell uptake the neutral red dye accumulates in lysosomes, the low pH in the lysosomal matrix of live cells causes the dye to becomes charged, allowing it to be retained in this organelle.[15] The dye was then extracted from the cells upon solubilisation and quantified. *Protocol:* a stock solution of neutral red was prepared in water (4 mg. mL^{-1}) and diluted 80-fold in the cell culture medium (RPMI). Prior to addition to the cells, the solution was heated to 37 ^{o}C and filtered (no. 1 Whatman paper). The growth medium was removed from the cells, the neutral red dye (100 µL) was added and the cells are incubated for 3 hours in the dark. Upon removal of the dye, the cells were rinsed with PBS (100 µL) and then the plates were shaken for 15 min with PBS (200 µL) for solubilisation. The dye concentration was quantified by measuring the absorbance at 450 nm. An IC_{50} value was determined and is defined as the concentration required to inhibit dye uptake by 50%. This was done using non-linear regression (Graphpad Prism). Goodness of fit was determined from the R^2 values of the curves and 95% confidence intervals. All cell experiments were performed in triplicate and repeated independently a minimum of two times.

Syntheses

***Caution!* No problems were encountered during this work, however heavy metal azides are known to be shock sensitive detonators, therefore it is essential that platinum azides compound are handled with care. The Pt-diazido complexes were synthesised and handled under dim lighting conditions.**

Synthesis of *cis*-N-heterocyclic starting materials

***Cis*-[Pt(I)$_2$(pyridine)$_2$] (8)**

K_2PtCl_4 (0.500 g, 1.21 mmol) was suspended in H_2O (38 mL) and KI (2.0 g, 12.05 mmol) was added and allowed to react with stirring for 1 h at room temperature. Pyridine (0.194 mL, 2.41 mmol) was added and the mixture was stirred at room temperature for 24 h. The dark yellow solid was isolated via filtration under suction and washed with ice cold water, ethanol and diethyl ether. The solid was then left to dry overnight under vacuum (Yield = 0.724 g, 99 %).

^{1}H-NMR (CD_2Cl_2, 400 MHz) δ: 8.84 (dd, $^3J_{195Pt1H}$ = 39.9 Hz, $^3J_{1H1H}$ = 6.7 Hz, 2H, H_o), 7.82 (t, $^3J_{1H1H}$ = 7.6 Hz, 1H, H_p), 7.34 (t, $^3J_{1H1H}$ = 6.7 Hz, 2H, H_m).

***Cis*-[Pt(Cl)$_2$(pyridine)$_2$]) (9)**

Cis-[Pt(I)$_2$(py)$_2$] (0.7229 g, 1.19 mmol) was suspended in H_2O (143 mL) and $AgNO_3$ (0.401 g, 2.36 mmol) was added. The mixture was allowed to react under reflux for 24 h at 60 ^{o}C, to yield a yellow precipitate (AgI). The AgI was removed by centrifugation, followed by filtration through celite on a frit, then NaCl (0.696 g, 11.91 mmol) was added and the reaction was carried out for 24 h at room temperature. The pale yellow solid was isolated by filtration and washed with cold water, ethanol and diethyl ether (Yield= 0.39 g, 77 %).

^{1}H-NMR ($CDCl_3$, 400 MHz) δ: 8.76 (dd, $^3J_{195Pt1H}$ = 36.5 Hz, $^3J_{1H1H}$ = 6.8 Hz, 2H, H_o), 7.84 (t, $^3J_{1H1H}$ =7.7 Hz, 1H, H_p), 7.33 (t, $^3J_{1H1H}$ =6.9 Hz, 2H, H_m).

***Cis*-[Pt(I)$_2$(2-picoline)$_2$] (10)**

K_2PtCl_4 (0.200 g, 0.48 mmol) was suspended in H_2O (110 mL), KI (0.800 g, 4.82 mmol) was added and the reaction was left to stir for 40 min at room temperature. 2-Picoline (0.095 mL, 0.96 mmol) was added and the reaction mixture was stirred for 24 h at 40 ^{o}C. The dark yellow solid was collected by filtering under vacuum and washed with cold water, ethanol and diethyl ether (Yield=0.268 g, 88%).

^{1}H-NMR (acetone-d_6, 600 MHz) δ: 9.26 (dd, $^3J_{195Pt1H}$ =31.5 Hz, $^3J_{1H1H}$ = 6.0 Hz, 1H, H_6 or $H_{6'}$), 9.12 (dd, $^3J_{195Pt1H}$ =31.4 Hz, $^3J_{1H1H}$ =5.9 Hz, 1H, $H_{6 \text{ or } 6'}$), 7.80 (t, $^3J_{1H1H}$ = 7.0 Hz, 1H, $H_{4 \text{ or } 4'}$), 7.79 (t, $^3J_{1H1H}$ = 7.0 Hz, 1H, $H_{4 \text{ or } 4'}$), 7.48 (m, $^3J_{1H1H}$ = 6.7 Hz, 2H, $H_{3 \text{ and } 3'}$), 7.37 (t, $^3J_{1H1H}$=6.8 Hz, 1H, $H_{5 \text{ or } 5'}$), 7.33 (t, $^3J_{1H1H}$ = 6.6 Hz, 1H, $H_{5 \text{ or } 5'}$), 3.34 (s, 3H, CH_3), 3.25 (s, 3H, CH_3).

***Cis*-[Pt(Cl)$_2$(2-picoline)$_2$] (11)**

Cis-[Pt(I)$_2$(2-pic)$_2$] (0.212 g, 0.34 mmol) was suspended in H_2O (54 mL), $AgNO_3$ (0.112 g, 0.66 mmol) was added and the reaction was stirred under reflux at 60 ^{o}C for 24 h. The AgCl formed was removed by filtration under suction using celite on a frit and also an inorganic membrane filter to ensure complete removal of traces of AgCl. NaCl (0.195 g, 3.34 mmol) was added and the reaction was left to stir for 24 h at ambient temperature. The pale yellow solid was isolated by filtration under suction and washed with cold water, ethanol and diethyl ether (Yield= 0.104 g, 68%).

^{1}H-NMR (acetone-d_6, 400 MHz) δ: 9.1 (broad peak, 2H, H_6), 7.8 (t, $^3J_{1H1H}$ = 7.8 Hz, 2H, H_4), 7.5 (d, $^3J_{1H1H}$ = 7.7 Hz, 2H, H_3), 7.3 (broad peak, 2H, H_5), 3.2 (s, 6H, CH_3).

Synthesis of mixed *trans*-N-heterocyclic starting materials

***Trans*-[Pt(Cl)$_2$(pyridine)(n-picoline)], where n = 2, 3 or 4**

Cis-[Pt(Cl)$_2$(py)$_2$] (0.200 g, 0.47 mmol) was suspended in H_2O (60 mL) and then n-picoline (4 eq, 0.176 g, 1.89 mmol in the case of 2-picoline; 2 eq, 0.044 g, 0.473 mmol for 3- and 4-picolines) was added and the solution was stirred at 75 °C overnight. When the solution turned colourless (~12 h for 2-picoline and 2 h for 3- and 4-picoline), the water was removed by rotary evaporation. The residual white solid was washed with diethyl ether to remove the excess ligand. Then, HCl (2 M, 1.3 mL) and H_2O (8 mL) were added and the reaction stirred for 4 days at 70 °C. The pale yellow solid was isolated by filtration and washed with water, ethanol and diethyl ether.

***Trans*-[Pt(Cl)$_2$(py)(2-pic)] (12)**

Yield = 0.155 g, 75%.

^{1}H-NMR (acetone-d_6, 600 MHz) δ: 8.94 (dd, $^3J_{195Pt1H}$= 16.0 Hz $^3J_{1H1H}$= 5.1 Hz, 2H, H_8), 8.87 (d, $^3J_{1H1H}$ = 5.5 Hz, 1H, H_6), 8.02 (t, $^3J_{1H1H}$ = 7.8 Hz, 1H, H_{10}), 7.84 (t, $^3J_{1H1H}$ = 7.7 Hz, 1H, H_4), 7.52 (m, 2H, H_9, H_3), 7.34 (t, $^3J_{1H1H}$ = 6.7 Hz, 1H, H_5), 3.25 (s, 3H, CH_3). ^{13}C-NMR (acetone-d_6, 150 MHz) δ: 161.9 (C_2), 154.4 (C_6), 154.08 (C_8), 139.4 (C_{10}), 139.0 (C_4), 126.8 (C_3), 126.0 (C_9), 123.2 (C_5), 26.3 ppm (C_{CH3}).

[ESI-MS]= [M+Na]$^+$,[2M+Na]$^+$ (*m/z*) Calc., 461.0, 899.0; Found, 460.9, 898.9.

***Trans*-[Pt(Cl)$_2$(py)(3-pic)] (13)**

Yield= 0.135 g, 65%.

^{1}H-NMR (acetone-d_6, 600 MHz) δ: 8.89 (d, $^3J_{195Pt1H}$= 13.2 Hz $^3J_{1H1H}$=6.8 Hz, 2H, H_8), 8.70 (d, $^3J_{195Pt1H}$= 13.5 Hz, H_2), 8.68 (d, $^3J_{1H1H}$= 5.9 Hz, 1H, H_6), 8.02 (t, $^3J_{1H1H}$=7.7 Hz, 1H, H_{10}), 7.83 (t, $^3J_{1H1H}$= 7.8 Hz, 1H, H_4), 7.52 (t, $^3J_{1H1H}$= 6.5 Hz, 2H, H_9), 7.39 (t, $^3J_{1H1H}$= 6.8 Hz, 1H, H_5), 2.41 (s, 3H, CH_3).

^{13}C-NMR (acetone-d_6, 150 MHz) δ: 155.3 (C_8), 155.2 (C_2), 152.5 (C_6), 141.1 (C_2), 140.5 (C_{10}), 136.22 (C_3), 127.1 (C_9), 126.4 (C_5), 19.0 (C_{CH3}).

[ESI-MS]= [M+Na]$^+$,[2M+Na]$^+$ (*m/z*) Calc., 461.0, 899.0; Found, 460.9, 898.9

***Trans*-[Pt(Cl)$_2$(py)(4-pic)] (14)**

Yield = 0.140 g, 67%.

^{1}H-NMR (acetone-d_6, 600 MHz) δ: 8.89 (dd, $^3J_{195Pt1H}$= 15.8 Hz, $^3J_{1H1H}$= 5.9 Hz, 2H, H_6), 8.69 (dd, $^3J_{195Pt1H}$= 15.2 Hz, $^3J_{1H1H}$= 6.5 Hz, 2H, H_2), 8.02 (t, $^3J_{1H1H}$=7.8 Hz, 1H, H_8), 7.51 (t, $^3J_{1H1H}$=6.7 Hz, 2H, H_7), 7.33 (d, $^3J_{1H1H}$=6.1 Hz, 2H, H_3), 2.47 (s, 3H, CH_3).

^{13}C-NMR (acetone-d_6, 150 MHz) δ: 155.3 (C_5), 154.4 (C_2), 152.9 (C_4), 140.5 (C_7), 127.8 (C_3), 127.1 (C6), 21.7 (C_{CH3}).

[ESI-MS]= [M+Na]$^+$,[2M+Na]$^+$ (*m/z*) Calc., 461.0, 899.0; Found, 460.9, 898.9

General procedure for synthesis of *trans*-[Pt(N_3)$_2$(py)(n-pic)] complexes

Trans-[Pt(Cl)$_2$(py)(n-pic)] (0.155 g, 0.35 mmol; 0.245 g, 0.56 mmol; 0.24 g, 0.55 mmol for 2-, 3- and 4-picoline, respectively) were suspended in H_2O (204 mL per 1 g). $AgNO_3$ (2 mol eq) was added and the reaction was carried out at 60 ^oC for 24 h. The AgCl was removed by filtration on celite on frit and also through an IM filter. NaN_3 (10 mol eq) was added and the mixture was allowed to stir for 24 h at 40 ^oC. The yellow solid was isolated and washed with cold water, ethanol and diethyl ether. Recrystallisation of these complexes was carried out by dissolving the solid in hot MeOH (50 ^oC, ~0.5 g/L) and then allowing the solution to stand at -20 ^oC.

***Trans*-[Pt(N_3)$_2$(py)(2-pic)] (15)**

Yield = 0.1029 g, 64%.

^{1}H-NMR (acetone-*d*$_6$, 400 MHz) δ: 9.07 (dd, $^3J_{1H1H}$= 6.0 Hz, $^3J_{195Pt1H}$ = 32.0 Hz, 1H, H_4), 8.77 (dd, $^3J_{1H1H}$ = 5.5 Hz, $^3J_{195Pt1H}$ = 39.0 Hz, 2H, H_5), 8.11 (t, $^3J_{1H1H}$= 7.5 Hz, 1H, H_7), 7.96 (t, $^3J_{1H1H}$= 7.8 Hz, 1H, H_2), 7.68 (d, $^3J_{1H1H}$= 7.8, 1H, H_1), 7.60 (t, $^3J_{1H1H}$= 6.8 Hz, 2H, H_6), 7.52 (t, $^3J_{1H1H}$= 7.0 Hz, 1H, H_3), 3.31 (s, CH_3).

^{13}C-NMR (acetone-*d*$_6$, 150 MHz) δ: 161.9 (C_2), 154.3 (C_4), 152.4 (C_8), 140.0 (C_{10}), 139.8 (C_4), 127.5 (C_3), 126.4 (C_9), 124.5 (C_5), 25.8 (C_{CH3}).

^{195}Pt-NMR (acetone-*d*$_6$, 129 MHz) δ : -2118

[ESI-MS]= [M+Na]$^+$ (*m/z*) Calc., 474.0; Found, 473.9.

***Trans*-[Pt(N_3)$_2$(py)(3-pic)] (16)**

Yield= 0.177 g, 70 %.

^{1}H-NMR (acetone-*d*$_6$, 600 MHz) δ: 8.84 (dd, $^3J_{1H1H}$= 5.1 Hz, $^3J_{195Pt1H}$= 32.0 Hz, 2H, H_8), 8.68 (s, 1H, H_2), 8.65 (d, $^3J_{1H1H}$= 5.8 Hz, 1H, H_6), 8.12 (t, $^3J_{1H1H}$= 7.4 Hz, 1H, H_{10}), 7.92 (d, $^3J_{1H1H}$= 7.8, 1H, H_4), 7.66 (t, $^3J_{1H1H}$= 6.9 Hz, 2H, H_9), 7.54 (t, $^3J_{1H1H}$= 7.1 Hz, 1H, H_5), 2.46 (s, 3H, CH_3).

^{195}Pt-NMR (acetone-*d*$_6$, 129 MHz) δ : -2120.

[ESI-MS]= [M+Na]$^+$ (*m/z*) Calc., 474.0; Found, 473.9.

Trans-[Pt(N_3)$_2$(py)(4-pic)] (17)

Yield = 0.167 g, 66%.

^{1}H-NMR (acetone-*d*$_6$, 600 MHz) δ: 8.85 (dd, $^3J_{1H1H}$=5.7 Hz, $^3J_{195Pt1H}$=30.0 Hz, 2H, H_6), 8.66 (dd, $^3J_{1H1H}$=5.8 Hz, 2H, H_2), 8.11 (t, $^3J_{1H1H}$=7.77 Hz, 1H, H_8), 7.65 (t, $^3J_{1H1H}$=6.5 Hz, 2H, H_7), 7.48 (d, $^3J_{1H1H}$= 5.6 Hz, 1H, H_2), 2.52 (s, 3H, CH_3).

^{195}Pt-NMR (acetone-*d*$_6$, 129 MHz) δ: -2117.

[ESI-MS]= [M+Na]$^+$ (*m/z*) Calc., 474.0; Found, 473.9.

General procedure for synthesis of *trans, trans, trans*-[Pt(N_3)$_2$(OH)$_2$(py)(n-pic)] complexes

Trans-[Pt(N_3)$_2$(py)(n-pic)] (0.084 g, 0.18 mmol; 0.138 g, 0.30 mmol; 0.108 g, 0.18 mmol for 2-, 3- and 4-picoline, respectively) was suspended in H_2O_2 (30% v/v, 70 mL per 1 g) and stirred at 45 ^oC for 3 hours. A bright yellow solution formed, which was filtered (IM) to remove any unreacted starting material or insoluble side-products. Then, the H_2O_2 was removed by lyophilization. To isolate the final product, the residual yellow precipitate was suspended in the minimum amount of warm ethanol (45 ^oC) and then it was allowed to precipitate upon addition of diethyl ether (3-fold) at -20 ^oC.

***Trans, trans, trans*-[Pt(N_3)$_2$(OH)$_2$(py)(2-pic)] (18)**

Yield = 0.023 g, 25%.

^{1}H-NMR (D_2O, 700 MHz) δ: 8.78 (d, $^3J_{1H1H}$=6.4 Hz, 1H, H_6), 8.73 (d, $^3J_{1H1H}$= 6.0 Hz, 2H, H_8), 8.26 (t, $^3J_{1H1H}$= 7.1 Hz, 1H, H_{10}), 8.06 (t, $^3J_{1H1H}$= 7.6 Hz, 1H, H_4), 7. 80 (t, $^3J_{1H1H}$= 6.5, 2H, H_9), 7.60 (d, $^3J_{1H1H}$=6.8 Hz, 1H, H_3), 7.56 (t, $^3J_{1H1H}$=7.0 Hz, 1H, H_5), 3.32 (s, 3H, CH_3).

^{13}C-NMR (D_2O, 175 MHz) δ: 163.3 (C_2), 152.8 (C_6), 149.8 (C_8), 143.2 (C_{10}), 142.3 (C_4), 130.1 (C_3), 127.6 (C_9), 124.8 (C_5), 22.9 (C_{CH3}).

^{195}Pt-NMR (D_2O, 129 MHz) δ: 1132.

[ESI-MS]= [M+Na]$^+$ (*m/z*) Calc., 508.0; Found, 508.1.

$\varepsilon_{297\ nm}$ =20,091 $M^{-1}cm^{-1}$, $\varepsilon_{268\ nm}$ =16,061 $M^{-1}cm^{-1}$ (H_2O).

***Trans, trans, trans*-[Pt(N_3)$_2$(OH)$_2$(py)(3-pic)] (19)**

Yield = 0.073 g, 49%.

^{1}H-NMR (D_2O, 600 MHz) δ: 8.83 (dd, $^3J_{1H1H}$= 5.9 Hz, $^3J_{195Pt1H}$= 32.0 Hz, 2H, H_8), 8.64 (s, 1H, H_2), 8.63 (d, $^3J_{1H1H}$=7.0 Hz, 1H, H_6), 8.31 (t, $^3J_{1H1H}$= 7.5 Hz, 1H, H_{10}), 8.14 (d, $^3J_{1H1H}$= 7.8, 1H, H_4), 7.85 (t, $^3J_{1H1H}$= 7.0 Hz, 2H, H_9), 7.72 (t, $^3J_{1H1H}$= 7.0 Hz, 1H, H_5), 2.56 (s, 3H, CH_3).

^{13}C-NMR (D_2O, 150 MHz): δ 150.9 (C_8), 150.7 (C_2), 148.0 (C_6), 144.6 (C_4), 144.1 (C_{10}), 139.6 (C_3), 128.7 (C_9), 127.9 (C_5), 19.3 (C_{CH3}).

^{195}Pt-NMR (D_2O, 129 MHz) δ: 954.

[ESI-MS]= [M+Na]$^+$ (*m/z*) Calc., 508.0; Found, 508.0.

$\varepsilon_{294\ nm}$ =18,258 $M^{-1}cm^{-1}$, $\varepsilon_{268\ nm}$ =13,467 $M^{-1}cm^{-1}$ (H_2O).

***Trans, trans, trans*-[Pt(N_3)$_2$(OH)$_2$(py)(4-pic)] (20)**

Crystals suitable for X-ray diffraction were obtained via suspension of **20** in warm ethanol and addition of diethyl ether (3-fold), followed by storage at -20 ^oC.

Yield = 0.074 g, 50%.

^{1}H-NMR (D_2O, 600 MHz) δ: 8.78 (dd, $^3J_{1H1H}$=5.9 Hz, $^3J_{195Pt1H}$= 48.0 Hz, 2H, H_6), 8.59 (d, dd, $^3J_{1H1H}$= 6.1 Hz, $^3J_{195Pt1H}$=52.0 Hz, 2H, H_2), 8.27 (t, $^3J_{1H1H}$= 6.9 Hz, 1H, H_8), 7.81 (t, $^3J_{1H1H}$= 6.4 Hz, 2H, H_7), 7.63 (d, $^3J_{1H1H}$= 6.1 Hz, 2H, H_3), 2.60 (s, 3H, CH_3).

^{13}C-NMR (D_2O, 150 MHz) δ: 158.7 (C_4), 150.0 (C_6), 148.9 (C_2), 143.1 (C_8), 128.5 (C_3), 127.7 (C_7), 21.1 (C_{CH3}).

^{195}Pt-NMR (D_2O, 129 MHz) δ: 957.

[ESI-MS]= [M+Na]$^+$ (*m/z*) Calc., 508.0; Found, 508.1.

$\varepsilon_{295\ nm}$ = 18,955 $M^{-1}cm^{-1}$, $\varepsilon_{258\ nm}$ = 12,535 $M^{-1}cm^{-1}$ (H_2O).

Syntheses of *bis trans*-N-heterocyclic complexes

***Trans*-[Pt(Cl)$_2$(3-pic)$_2$] (21)**

K_2PtCl_4 (0.700 g, 1.69 mmol) was dissolved in H_2O (55 mL) and 3-picoline (50 eq, 7.51 mL) was added. The solution was allowed to react under reflux at 100 ^oC for 24 h. The water and excess ligand were evaporated and the white residue was washed with diethyl ether. Then HCl (2 M, 24 mL) was added and the reaction proceeded for 48 h at 85 ^oC. The yellow precipitate was collected by filtration (Yield = 0.706 g, 93%).

^1H-NMR ($CDCl_3$, 400 MHz) δ: 8.72 (s, 2H, H_2), 8.69 (d, $^3J_{1H1H}$= 5.5 Hz, 2H, H_6), 7.57 (d, $^3J_{1H1H}$= 8.0 Hz, 2H, H_4), 7.19 (t, $^3J_{1H1H}$= 6.7 Hz, 2H, H_5), 2.37 (s, 6H, CH_3).

***Trans*-[Pt(N$_3$)$_2$(3-pic)$_2$] (22)**

Trans-[Pt(Cl)$_2$(3-pic)$_2$] (0.700 g, 1.55 mmol) was dissolved in DMF (30 mL), NaN_3 (20 eq, 2.013 g) was added and the reaction was stirred for 4 d at 35 ^oC. Work up of the reaction was carried out by the addition of diethyl ether (60 mL) and precipitation of the solid at -20 ^oC (Yield= 0.400 g, 55%).

^1H-NMR ($CDCl_3$, 400 MHz) δ: 8.63 (s, 2H, H_2), 8.61 (d, $^3J_{1H1H}$= 6.5 Hz, 2H, H_6), 7.66 (d, $^3J_{1H1H}$= 7.8 Hz, 2H, H_4), δ= 7.33 (t, $^3J_{1H1H}$= 6.8 Hz, 2H, H_5), 2.43 (s, 6H, CH_3).

[ESI-MS]= $[M+Na]^+$ (*m/z*) Calc., 488.1; Found, 488.0.

***Trans, trans, trans*-[Pt(N$_3$)$_2$(OH)$_2$(3-pic)$_2$] (23)**

Trans-[Pt(N$_3$)$_2$(3-pic)$_2$] (0.350 g, 0.75 mmol) was suspended in H_2O_2 (30% v/v, 51 mL) and allowed to react at 50 ^oC for 5 h, at which point the solution turned transparent. The solution was filtered using an IM filter to remove unreacted starting material or insoluble side products. Then the H_2O_2 was removed by lyophilization. To isolate the final product, the residual yellow precipitate was suspended in the minimum amount of warm ethanol (45 ^oC) and then it was allowed to precipitate upon addition of diethyl ether (3-fold) at -20 ^oC. Crystals suitable for X-ray diffraction were obtained by slow evaporation of a solution of the complex in MeOH/DCM at room temperature.

^1H-NMR (D_2O, 500 MHz) δ: 8.60 (s, 2H, H_2), 8.59 (d, $^3J_{1H1H}$= 5.5 Hz, 2H, H_6), 8.10 (d, $^3J_{1H1H}$= 7.8 Hz, 2H, H_4), 7.68 (t, $^3J_{1H1H}$= 7.1 Hz, 2H, H_5), 2.52 (s, 6H, CH_3).

^{13}C-NMR (D_2O, 125 MHz) δ: 149.7 (C_1), 147.0 (C_6), 143.6 (C_4), 138.7 (C_3), 127.0 (C_5), 18.4 (C_{CH3}).

^{195}Pt-NMR (D_2O, 107 MHz) δ: 971.

[ESI-MS]= $[M+Na]^+$ (*m/z*) Calc., 522.1; Found, 521.9.

$\varepsilon_{295\ nm}$ =19025 $M^{-1}cm^{-1}$, $\varepsilon_{267\ nm}$ =13766 $M^{-1}cm^{-1}$ (H_2O).

***Trans*-[Pt(Cl)$_2$(4-pic)$_2$] (24)**

K_2PtCl_4 (0.800 g, 1.93 mmol) was completely dissolved in H_2O (24 mL), 4-picoline (20 eq, 3.75 mL) was added and allowed to react at 95 ^oC for 1 h until the solution turned colourless. The solvent was rotary evaporated to dryness and the residual white solid was washed with cold diethyl ether, suspended in HCl (2 M, 27 mL) and allowed to react for 12 h at 85 ^oC. The solution was cooled on ice, filtered and the yellow precipitate product washed with cold solvents (H_2O, ethanol, diethyl ether) (Yield = 0.697 g, 80%).

^1H-NMR ($CDCl_3$, 400 MHz) δ: 8.71 (d, $^3J_{1H1H}$= 6.8 Hz, 4H, H_2), 7.11 (d, $^3J_{1H1H}$= 6.8 Hz, 4H, H_3), 2.41 (s, 6H, CH_3).

***Trans*-[Pt(N_3)$_2$(4-pic)$_2$] (25)**

Trans-[Pt(Cl)$_2$(4-pic)$_2$] (0.690 g, 1.53 mmol) was dissolved in DMF (35 mL), then NaN_3 (20 eq, 1.282 g) was added and the mixture was allowed to react for 4 d at room temperature in the dark. The solution was then placed at -20 ^oC, for 2 d after which the yellow product was isolated by filtration. A second crop of precipitate was collected by the addition an equal volume of diethyl ether to the filtrate, followed by storage at -20 ^oC for a further 2d. The precipitates were combined, washed with cold solvents (H_2O, ethanol, ether) to give the product as a yellow solid (Yield = 0.605 g, 85%).

^{1}H-NMR (acetone-d_6, 400 MHz) δ: 8.65 (dd, $^3J_{195Pt1H}$= 34.0 Hz, $^3J_{1H1H}$= 6.6 Hz, 4H, H_2), 7.47 (d, $^3J_{1H1H}$= 6.0 Hz, 4H, H_3), 2.51 (s, 6H, CH_3).

[ESI-MS]= [M+Na] $^+$ (*m/z*) Calc., 488.1; Found, 488.0.

^{195}Pt NMR (d_6-acetone, 129 MHz) *δ*: - 2118.

***Trans*-[Pt(N_3)$_2$(OH)$_2$(4-pic)$_2$] (26)**

Trans-[Pt(N_3)$_2$(4-pic)$_2$] (0.600 g, 1.29 mmol) was suspended in H_2O_2 (30% v/v, 150 ml) and stirred for 20 h at 50 ^oC, to give a yellow solution which was lyophilized. Crystallisation was carried out by dissolving the solid in warm methanol (2 mL, 55 ^oC) and then adding diethyl ether (20 mL). The mixture was placed at -20 ^oC to give the title compound as a yellow solid. (Yield= 0.193 g, 30%). Crystals suitable for X-ray diffraction were obtained by the slow evaporation at ambient temperature of a methanolic solution of **26**.

^{1}H-NMR (D_2O, 400 MHz) δ: 8.57 (dd, $^3J_{195Pt1H}$= 26.8 Hz, $^3J_{1H1H}$= 6.8 Hz, 4H, H_2), 7.62 (d, $^3J_{1H1H}$= 6.2 Hz, 4H, H_3), 2.60 (s, 6H, CH_3).

^{13}C-NMR (D_2O, 150 MHz) δ: 156.6 (C_4), 148.9 (C_2), 128.5 (C_3), 21.3 (C_{CH3}).

^{195}Pt-NMR (D_2O, 129 MHz): δ: 964.

[ESI-MS]= [M+Na] $^+$ (*m/z*) Calc., 522.1; Found, 522.1.

$\varepsilon_{293\ nm}$ =15,815 $M^{-1}cm^{-1}$, $\varepsilon_{256\ nm}$ =12,689 $M^{-1}cm^{-1}$ (H_2O).

***Trans*-[Pt(Cl)$_2$(tz)$_2$] (27)**

K_2PtCl_4 (0.800 g, 1.93 mmol) was completely dissolved in H_2O (16 mL) and thiazole (14 eq, 2.29 mL) was added. The solution was allowed to react, under reflux, at 100 ^oC for three hours until the mixture turned into a transparent yellow solution (an orange precipitate is formed as an intermediate in the reaction). The solvent was rotary evaporated to dryness and the residual yellow solid was suspended in HCl (2 M, 24 mL) and allowed to react overnight (14 hours) at 85 ^oC. The yellow precipitate was filtered, washed with water, ethanol and diethyl ether (Yield = 0.750 g, 89%). The product was re-crystallised from hot 0.1 M HCl in ethanol, affording yellow crystals suitable for X-ray diffraction.

^{1}H-NMR (acetone-d_6, 400 MHz) δ: 9.58 (dd, $^3J_{1H1H}$= 2.4 Hz, $^3J_{195Pt1H}$= 27.0 Hz, 2H, H_2), 8.32 (d, $^3J_{1H1H}$= 3.4 Hz, 2H, H_5), 7.88 (t, $^3J_{1H1H}$= 2.9 Hz, 3.5Hz, 2H, H_5). ^{13}C (150.9 MHz, d_6-acetone) *δ*: 158.5 (C_2), 144.8 (C_5), 120.8 (C_4).

[ESI-MS]= [M+Na]$^+$, [2M+Na]$^+$ (*m/z*) Calc., 458.9, 894.8; Found, 458.8, 894.7

***Trans*-[Pt(N_3)$_2$(tz)$_2$] (28)**

Trans-[Pt(Cl)$_2$(tz)$_2$] (0.700 g, 1.61 mmol) was completely dissolved in DMF (30 mL) and NaN_3 (20 eq, 2.087 g) was added. The reaction was allowed to proceed for 6 d at room temperature in the dark and then placed in the freezer (-20 ^oC) overnight. Diethyl ether (30ml) was added and the solution returned

to the freezer. After 6 h yellow crystals and a white precipitate formed, the crystals were isolated by filtration and rinsed with minimal cold H_2O to remove the white precipitate by dissolution. (Yield= 0.411 g, 57%). Crystals suitable for x-ray crystallography were grown by diffusion of diethyl ether into an acetone solution of the product.

^1H-NMR (acetone-d_6, 400 MHz) δ: 9.57 (dd, $^3J_{1H1H}$= 2.05 Hz, $^3J_{195Pt1H}$= 29.0 Hz, 2H, H_2), 8.26 (d, $^3J_{1H1H}$= 3.7 Hz, 2H, H_5), 8.04 (t, $^3J_{1H1H}$= 2.8 Hz, 3.5 Hz, 2H, H_4).

^{13}C (150.9 MHz, d_6-acetone) *δ*: 158.4 (C_2), 144.2 (C_5), 122.6 (C_4).

[ESI-MS]= [M+Na] $^+$ (*m/z*) Calc., 488.0; Found, 488.0.

***Trans, trans, trans*-[Pt(N$_3$)$_2$(OH)$_2$(tz)$_2$] (29)**

Trans-[Pt(N_3)$_2$(tz)$_2$] (0.400 g, 0.89 mmol) was suspended in H_2O_2 (30% v/v, 45 mL) and allowed to react at 50 ^oC for 6 hours. The yellow solution was IM filtered and then the H_2O_2 removed by freeze drying. Cold acetone was used to rinse the residual yellow solid from the glass and the product was filtered under suction and washed with cold water, ethanol and ether (Yield= 0.215 g, 50%).

^1H-NMR (D_2O, 400 MHz) δ: 9.58 (dd, $^3J_{1H1H}$= 2.0 Hz, $^3J_{195Pt1H}$= 20.3 Hz, 2H, H_2), 8.30 (dd, $^3J_{1H1H}$= 3.8 Hz, $^3J_{195Pt1H}$= 15.7 Hz, 2H, H_5), 8.07 (t, $^3J_{1H1H}$= 2.93 Hz, 2H, H_4).

^{13}C-NMR (D_2O, 150 MHz) δ: 157.8 (C_2), 140.3 (C_5), 123.8 (J_{CPt} 35 Hz, C_4).

^{195}Pt-NMR (D_2O, 129 MHz) δ: 977.

$\varepsilon_{295\ nm}$ =16,750 $M^{-1}cm^{-1}$ (H_2O).

^{14}N NMR (D_2O, dioxane, 37.5^oC) *δ*: 229 ($W_{½}$ 86 Hz), 169 ($W_{½}$ 400 Hz).

ESI-MS (H_2O) *m/z* (**29**): 1472.9500 (3%, [3.(**29**)+Na]$^+$, calcd. 1472.9456), 1450.9690 (1%, [3.(**29**)+H]$^+$, calcd. 1450.9637), 988.9613 (100%, [2.(29)+Na]$^+$, calcd. 988.9591), 966.9796 (8%, [2.(**29**)+H]$^+$,calcd. 966.9771), 505.9766 (33%, [(**29**)+Na]$^+$,calcd. 505.9751).

MS/MS (505.9766, [**29**+Na]$^+$): 378.9689 (loss of N_3 and tz; [Pt(N_3)(OH)$_2$(tz)+Na]$^+$, calcd. 378.9673), 350.9623 (loss of N_3, N_2 and tz; [Pt(N)(OH)$_2$(tz)+Na]$^+$, calcd. 350.9612).

***Trans*-[Pt(Cl)$_2$(1-methylimidazole)$_2$] (30)**

K_2PtCl_4 (0.5 g, 1.21 mmol) was dissolved in H_2O (15 mL), 1-methylimidazole (7 eq, 0.672 mL, 8.43 mmol) was added and the solution was stirred at 90 ^oC. Precipitation of a yellow intermediate was observed and the solution turned colourless after 120 min. Water was removed by rotary evaporation to leave a yellow solid and excess ligand. HCl (2 M, 15 mL) was then added and the reaction was allowed to proceed for 48 h at 85 ^oC, to give a yellow solid which was isolated by filtration and washed with water, ethanol and diethyl ether (Yield= 0.366 g, 71%).

^1H-NMR (acetone-d_6, 400 MHz) δ: 8.13 (s, 2H, H_2), 7.38 (t, $^3J_{1H1H}$ = 1.3 Hz, 2H, H_4), 7.11 (t, $^3J_{1H1H}$= 1.6 Hz, 2H, H_5), 3.86 (s, 6H, CH_3).

[ESI-MS]= [M+Na]$^+$, [2M+Na]$^+$ (*m/z*) Calc., 453.0, 883.0; Found, 452.9, 882.9

***Trans*-[Pt(N$_3$)$_2$(1-methylimidazole)$_2$] (31)**

Trans-[Pt(Cl)$_2$(mim)$_2$] (0.350 g, 0.82 mmol) was dissolved in DMF (7 mL) and NaN_3 (20 eq, 1.057 g) was suspended in the solution. The reaction was allowed to proceed for 4 days at 35 ^oC. Work up of the reaction was carried out by the addition of diethyl ether (60 mL) and precipitation of the solid at -20 ^oC (Yield= 0.110 g, 30%).

^1H-NMR (MeOH-d_4, 400 MHz) δ: 8.03 (s, 2H, H_2), 7.19 (s, 4H, H_4, H_5), 3.82 (s, 6H, CH_3).

[ESI-MS]= [M+Na] $^+$ (*m/z*) Calc., 466.1; Found, 465.9.

***Trans*-[Pt(OH)$_2$(N$_3$)$_2$(1-methylimidazole)$_2$] (32)**

Trans-[Pt(N$_3$)$_2$(mim)$_2$] (0.106 g, 0.24 mmol) was suspended in H_2O_2 (30% v/v, 9.2 mL) and the reaction was heated to 50 ^oC, until the yellow solution became transparent (1.5 h). Removal of the solvent was carried out via lyophilisation after the solution was IM-filtered. Recrystallisation in a methanol/ DCM solution allowed the growth of crystals suitable for their study by X-ray diffraction (Yield= 0.050 g, 44%).

^{1}H-NMR (D_2O, 500 MHz) δ: 8.22 (s, 2H, H1), 7.35 (t, $^3J_{1H1H}$= 1.5 Hz, 2H, H2), 7.32 (t, $^3J_{1H1H}$= 1.6 Hz, 2H, H3), 3.92 (s, 6H, CH_3).

^{13}C-NMR (D_2O, 125 MHz) δ: 137.48 (C_2), 125.54 (C_4), 123.05 (C_5), 35.59 (C_{CH3}).

^{195}Pt-NMR (D_2O, 107 MHz) δ: 987.

[ESI-MS]= [M+Na]$^+$ (*m/z*) Calc., 500.0; Found, 499.9. $\varepsilon_{290\ nm}$ =18307 $M^{-1}cm^{-1}$ (H_2O).

X-ray crystal data for complexes 28, 20, 23 26 and 32

Table S1 Crystal data and structure refinement for 28

Identification code	28
Empirical formula	$C_6H_6N_8PtS_2$
Formula weight	449.40
Temperature/K	100(2)
Crystal system	triclinic
Space group	P-1
a/Å	3.79960(10)
b/Å	8.1562(3)
c/Å	9.0053(3)
α/°	88.995(3)
β/°	86.555(3)
γ/°	76.997(3)
Volume/Å^3	271.427(16)
Z	1
ρ_{calc}g/cm^3	2.749
μ/mm^{-1}	13.298
F(000)	208.0
Crystal size/mm^3	0.32 × 0.08 × 0.04 yellow block
Radiation	MoKα (λ = 0.71073)
2Θ range for data collection/°	6.83 to 58.122
Index ranges	-5 ≤ h ≤ 4, -11 ≤ k ≤ 11, -11 ≤ l ≤ 12
Reflections collected	3931
Independent reflections	1314 [R_{int} = 0.0278, R_{sigma} = 0.0286]
Data/restraints/parameters	1314/0/79
Goodness-of-fit on F^2	1.080
Final R indexes [I>=2σ (I)]	R_1 = 0.0175, wR_2 = 0.0392
Final R indexes [all data]	R_1 = 0.0175, wR_2 = 0.0392
Largest diff. peak/hole / e Å^{-3}	0.76/-1.37

Table S2 Bond Lengths for 28

Atom	Atom	Length/Å	Atom	Atom	Length/Å
Pt1	N1^1	2.014(3)	C2	S3	1.699(3)
Pt1	N1	2.014(3)	S3	C4	1.712(4)
Pt1	N6^1	2.033(3)	C4	C5	1.365(5)
Pt1	N6	2.033(3)	N6	N7	1.213(4)
N1	C2	1.322(4)	N7	N8	1.156(4)
N1	C5	1.388(4)			

Table S3 Bond Angles for 28

Atom	Atom	Atom	Angle/°	Atom	Atom	Atom	Angle/°
N1[1]	Pt1	N1	180.00(16)	C5	N1	Pt1	122.0(2)
N1[1]	Pt1	N6[1]	86.62(11)	N1	C2	S3	113.5(3)
N1[1]	Pt1	N6	93.38(11)	C2	S3	C4	91.09(17)
N1	Pt1	N6	86.62(11)	C5	C4	S3	109.5(3)
N1	Pt1	N6[1]	93.38(11)	C4	C5	N1	114.4(3)
N6	Pt1	N6[1]	180.0	N7	N6	Pt1	123.4(2)
C2	N1	Pt1	126.4(2)	N8	N7	N6	174.8(4)
C2	N1	C5	111.4(3)				

[1]1-X,1-Y,1-Z

Table S4. X-ray crystal data for complexes 20, 23 26 and 32

CCDC: 1904755 (**28**); 1904751 (**20**), 1904753 (**26**), 1904752 (**23**), 1904754 (**32**), respectively

Complex	20	23·2CH$_3$OH	26·2CH$_3$OH†	32·0.5H$_2$O
**Empirical formula**	$C_{11}H_{14}N_8O_2Pt$	$C_{14}H_{24}N_8O_4Pt$	$C_{14}H_{16}N_8O_4Pt$	$C_8H_{16}N_{10}O_3Pt$
**Formula weight**	485.39	563.50	555.44	495.40
**Temperature/K**	100(2)	150(2)	100(2)	150(2)
**Crystal system**	monoclinic	monoclinic	triclinic	triclinic
**Space group**	$P2_1/n$	$P2_1/n$	P-1	P-1
**a/Å**	9.6152(3)	7.09413(13)	9.4521(2)	8.4653(5)
**b/Å**	9.1982(3)	11.18048(20)	9.5887(2)	8.7128(6)
**c/Å**	17.2275(5)	12.3270(2)	12.0611(2)	10.9563(4)
**α/°**	90	90	97.817(2)	71.076(5)
**β/°**	92.473(3)	90.6038(17)	105.393(2)	79.831(4)
**γ/°**	90	90	114.191(2)	84.892(6)
**Volume/Å^3**	1522.21(8)	977.67(3)	923.38(4)	751.97(8)
**Z**	4	2	2	2
**ρ_{calc}g/cm^3**	2.118	1.914	1.998	2.188
**μ/mm^{-1}**	9.238	7.214	7.637	9.361
**F(000)**	920.0	548.0	532.0	472.0
**Crystal size/mm^3**	0.408 × 0.29 × 0.137 colourless block	0.26 × 0.26 × 0.06 colourless block	0.562 × 0.35 × 0.244 yellow block	0.2 × 0.2 × 0.06
**2Θ range for data collection/°**	6.132 to 59.514	6.596 to 62.906	6.02 to 64.518	5.802 to 59.406
**Index ranges**	-13 ≤ h ≤ 9, -11 ≤ k ≤ 9, -23 ≤ l ≤ 23	-10 ≤ h ≤ 9, -16 ≤ k ≤ 15, -17 ≤ l ≤ 18	-14 ≤ h ≤ 13, -14 ≤ k ≤ 14, -17 ≤ l ≤ 17	-10 ≤ h ≤ 10, -11 ≤ k ≤ 9, -14 ≤ l ≤ 14
**Reflections collected**	8362	10718	19134	6671
**Independent reflections**	3790 [R_{int} = 0.0452, R_{sigma} = 0.0487]	3003 [R_{int} = 0.0268, R_{sigma} = 0.0259]	6084 [R_{int} = 0.0218, R_{sigma} = 0.0222]	3708 [R_{int} = 0.0408, R_{sigma} = 0.0534]
**Data/restraints/parameters**	3790/1/202	3003/0/128	6084/3/251	3708/3/209
**Goodness-of-fit on F^2**	1.046	1.052	1.134	1.021
**Final R indexes [I>=2σ (I)]**	R_1 = 0.0549, wR_2 = 0.1431	R_1 = 0.0185, wR_2 = 0.0409	R_1 = 0.0228, wR_2 = 0.0547	R_1 = 0.0396, wR_2 = 0.1000

† The asymmetric unit contains two 'half' Pt complexes where the Pt of each complex sits on an inversion centre and two solvent methanols (see later). The complexes are composed of two hydroxide, two azides and two 4-methyl pyridines. Four complete complexes and eight methanols in

the unit cell. One of the methanols (O1-C2) bridges between the OHs of the different complexes. The other methanol (O3 C4) bridges between this methanol and other symmetry related complexes. It was not possible to differentiate the C from the O from examination of the thermal ellipsoids or from the hydrogen bonding pattern so the oxygen and the carbon of that methanol were modelled as 50% occupancy at either position. The hydrogens (O100 and O200) were located on the OH ligands on each crystallographically independent Pt complex but refined with restraints. Any attempt to place hydrogens on the bridging solvent modelled at O3-O4 leads to alerts for short contacts. A model was constructed where the atoms of methanol (O3-O4) were modelled at 50% occupancy of a C and O so the methanol sits 50% one way and 50% the other way round. This makes it difficult to put hydrogens on the bridging methanol O1-C2 as half the time, O1 would be donating a hydrogen bond to C4. This means hydrogens cannot be placed on methanol O1-C2, leading to several crystallographic "B" alerts for a singly bonded carbons with no hydrogens.

Table S5: Bond lengths (Å) and angles (°) for complex **20**.

Bond Lengths for complex 20

Atom	Atom	Length/Å	Atom	Atom	Length/Å
Pt1	O1	2.011(7)	C102	C103	1.357(12)
Pt1	O2	2.011(6)	C103	C104	1.370(13)
Pt1	N1	2.021(8)	C104	C105	1.390(15)
Pt1	N4	2.038(7)	C104	C107	1.503(13)
Pt1	N101	2.005(8)	C105	C106	1.379(14)
Pt1	N201	2.023(8)	N201	C202	1.364(11)
N1	N2	1.217(11)	N201	C206	1.350(11)
N2	N3	1.150(11)	C202	C203	1.380(12)
N4	N5	1.236(11)	C203	C204	1.384(13)
N5	N6	1.139(12)	C204	C205	1.395(15)
N101	C102	1.373(11)	C205	C206	1.387(13)
N101	C106	1.365(10)			

Bond Angles for complex 20

Atom	Atom	Atom	Angle/˚	Atom	Atom	Atom	Angle/˚
O1	Pt1	N1	86.7(3)	C102	N101	Pt1	121.1(5)
O1	Pt1	N4	92.0(3)	C106	N101	Pt1	121.5(6)
O1	Pt1	N201	90.1(2)	C106	N101	C102	117.5(8)
O2	Pt1	O1	176.5(2)	C103	C102	N101	121.5(8)
O2	Pt1	N1	91.2(3)	C102	C103	C104	121.7(9)
O2	Pt1	N4	90.2(3)	C103	C104	C105	117.6(8)
O2	Pt1	N201	92.6(2)	C103	C104	C107	123.2(10)
N1	Pt1	N4	178.1(3)	C105	C104	C107	119.2(9)
N1	Pt1	N201	89.4(3)	C106	C105	C104	119.9(8)
N101	Pt1	O1	90.0(2)	N101	C106	C105	121.8(8)
N101	Pt1	O2	87.2(2)	C202	N201	Pt1	121.8(6)
N101	Pt1	N1	91.6(3)	C206	N201	Pt1	119.5(6)
N101	Pt1	N4	89.9(3)	C206	N201	C202	118.8(8)
N101	Pt1	N201	179.1(3)	N201	C202	C203	121.6(8)
N201	Pt1	N4	89.2(3)	C202	C203	C204	119.8(8)
N2	N1	Pt1	118.3(6)	C203	C204	C205	118.6(9)
N3	N2	N1	175.0(10)	C206	C205	C204	119.4(9)
N5	N4	Pt1	117.2(6)	N201	C206	C205	121.8(9)
N6	N5	N4	176.7(10)				

Table S6: Bond lengths (Å) and angles (°) for complex **23**·$2CH_3OH$

Bond Lengths for 23·$2CH_3OH$

Atom	Atom	Length/Å	Atom	Atom	Length/Å
Pt1	O1[1]	2.0053(19)	C2	C3	1.376(3)
Pt1	O1	2.0054(19)	C3	C4	1.387(4)
Pt1	N1	2.034(2)	C4	C5	1.399(3)
Pt1	N1[1]	2.034(2)	C5	C6	1.386(4)
Pt1	N8	2.048(2)	C5	C7	1.499(4)
Pt1	N8[1]	2.048(2)	N8	N9	1.217(3)
N1	C2	1.346(3)	N9	N10	1.147(3)
N1	C6	1.352(3)	O11	C12	1.412(3)

[1]1-X,1-Y,2-Z

Bond Angles for 23·$2CH_3OH$

Atom	Atom	Atom	Angle/°	Atom	Atom	Atom	Angle/°
O1[1]	Pt1	O1	180.0	N8	Pt1	N8[1]	180.0
O1	Pt1	N1[1]	88.09(8)	C2	N1	Pt1	119.18(17)
O1[1]	Pt1	N1[1]	91.91(8)	C2	N1	C6	120.0(2)
O1	Pt1	N1	91.91(8)	C6	N1	Pt1	120.80(16)
O1[1]	Pt1	N1	88.09(8)	N1	C2	C3	120.8(2)
O1[1]	Pt1	N8	92.27(7)	C2	C3	C4	119.5(2)
O1	Pt1	N8[1]	92.27(7)	C3	C4	C5	120.1(2)
O1	Pt1	N8	87.73(7)	C4	C5	C7	122.0(2)
O1[1]	Pt1	N8[1]	87.73(7)	C6	C5	C4	117.1(2)
N1	Pt1	N1[1]	180.0	C6	C5	C7	120.9(2)
N1	Pt1	N8[1]	89.47(8)	N1	C6	C5	122.4(2)
N1[1]	Pt1	N8[1]	90.53(8)	N9	N8	Pt1	115.56(16)
N1	Pt1	N8	90.53(8)	N10	N9	N8	174.5(2)
N1[1]	Pt1	N8	89.47(8)				

[1]1-X,1-Y,2-Z

Table S7: Bond lengths (Å) and angles (°) for complex **26**·2CH_3OH. Two platinum centres were found and they are represented as Pt1 and Pt2.

Table S7a: Bond Lengths for 26·2CH_3OH**.**

Atom	Atom	Length/Å	Atom	Atom	Length/Å
Pt1	O100	1.997(2)	N101	C106	1.350(4)
Pt1	O100[1]	1.997(2)	N209	N210	1.145(4)
Pt1	N101[1]	2.029(3)	C105	C106	1.382(5)
Pt1	N101	2.029(3)	C105	C104	1.391(5)
Pt1	N108[1]	2.045(3)	C202	C203	1.379(5)
Pt1	N108	2.045(3)	C206	C205	1.379(5)
Pt2	O200[2]	1.998(2)	C102	C103	1.383(4)
Pt2	O200	1.998(2)	C204	C203	1.393(5)
Pt2	N201[2]	2.031(3)	C204	C207	1.500(5)
Pt2	N201	2.031(3)	C204	C205	1.388(5)
Pt2	N208[2]	2.049(3)	C107	C104	1.497(5)
Pt2	N208	2.049(3)	C103	C104	1.391(5)
N201	C202	1.349(4)	N108	N109	1.226(4)
N201	C206	1.351(4)	N109	N110	1.151(4)
N208	N209	1.212(4)	C3	O4	1.435(5)
O3	C4	1.435(5)	O1	C2	1.465(4)
N101	C102	1.343(4)			

[1]1-X,-Y,-Z; [2]1-X,-Y,1-Z

Table S7b: Bond Angles for 26·2CH_3OH

Atom	Atom	Atom	Angle/˚	Atom	Atom	Atom	Angle/˚
O100	Pt1	O100[1]	180.0	N201	Pt2	N208	89.90(11)
O100[1]	Pt1	N101	90.33(10)	N201[2]	Pt2	N208[2]	89.90(11)
O100	Pt1	N101	89.67(10)	N208[2]	Pt2	N208	180.0
O100[1]	Pt1	N101[1]	89.67(10)	C202	N201	Pt2	119.5(2)
O100	Pt1	N101[1]	90.33(10)	C202	N201	C206	119.2(3)
O100[1]	Pt1	N108[1]	91.38(11)	C206	N201	Pt2	121.2(2)
O100	Pt1	N108[1]	88.62(11)	N209	N208	Pt2	115.5(2)
O100	Pt1	N108	91.38(11)	C102	N101	Pt1	120.3(2)
O100[1]	Pt1	N108	88.62(11)	C102	N101	C106	119.6(3)
N101[1]	Pt1	N101	180.0	C106	N101	Pt1	120.1(2)
N101[1]	Pt1	N108	89.25(11)	N210	N209	N208	174.6(4)
N101	Pt1	N108[1]	89.25(11)	C106	C105	C104	120.3(3)
N101[1]	Pt1	N108[1]	90.75(11)	N201	C202	C203	121.4(3)
N101	Pt1	N108	90.75(11)	N201	C206	C205	121.1(3)
N108[1]	Pt1	N108	180.0	N101	C102	C103	121.2(3)
O200[2]	Pt2	O200	180.0	N101	C106	C105	121.2(3)
O200	Pt2	N201	89.94(10)	C203	C204	C207	121.0(3)
O200	Pt2	N201[2]	90.06(10)	C205	C204	C203	116.9(3)
O200[2]	Pt2	N201	90.06(10)	C205	C204	C207	122.1(3)
O200[2]	Pt2	N201[2]	89.94(10)	C202	C203	C204	120.5(3)
O200	Pt2	N208	90.67(10)	C102	C103	C104	120.4(3)
O200	Pt2	N208[2]	89.33(10)	C105	C104	C107	120.9(3)
O200[2]	Pt2	N208	89.33(10)	C105	C104	C103	117.3(3)
O200[2]	Pt2	N208[2]	90.67(10)	C103	C104	C107	121.8(3)
N201[2]	Pt2	N201	180.00(17)	C206	C205	C204	120.9(3)
N201[2]	Pt2	N208	90.10(11)	N109	N108	Pt1	115.3(2)
N201	Pt2	N208[2]	90.10(11)	N110	N109	N108	175.8(3)

[1]1-X,-Y,-Z; [2]1-X,-Y,1-Z

Table S8: Bond lengths (Å) and angles (°) for complex **32**·$0.5H_2O$. Two independent platinum centres were found and they are represented as Pt1 and Pt2.

Bond Lengths for 32·$0.5H_2O$

Atom	Atom	Length/Å	Atom	Atom	Length/Å
Pt1	N1	1.991(5)	Pt2	$N11^2$	2.004(5)
Pt1	$N1^1$	1.991(5)	Pt2	N11	2.004(5)
Pt1	O1	1.997(4)	Pt2	N17	2.056(5)
Pt1	$O1^1$	1.997(4)	Pt2	$N17^2$	2.056(5)
Pt1	$N7^1$	2.051(5)	Pt2	$O2^2$	2.009(3)
Pt1	N7	2.051(5)	Pt2	O2	2.009(3)
N1	C2	1.328(7)	N11	C12	1.315(8)
N1	C5	1.392(7)	N11	C15	1.387(7)
C2	N3	1.327(7)	C12	N13	1.337(10)
N3	C4	1.369(7)	N13	C14	1.374(7)
N3	C6	1.475(8)	N13	C16	1.468(8)
C4	C5	1.336(9)	C14	C15	1.340(9)
N7	N8	1.194(8)	N17	N18	1.204(7)
N8	N9	1.169(9)	N18	N19	1.132(8)

^{1}-X,1-Y,2-Z; ^{2}-X,1-Y,1-Z

Bond Angles for 32·0.5H_2O

Atom	Atom	Atom	Angle/˚	Atom	Atom	Atom	Angle/˚
N1	Pt1	N1[1]	180.0	N11[2]	Pt2	N11	180.0
N1[1]	Pt1	O1[1]	89.86(17)	N11[2]	Pt2	N17[2]	89.78(19)
N1	Pt1	O1	89.86(17)	N11	Pt2	N17[2]	90.22(19)
N1[1]	Pt1	O1	90.14(17)	N11	Pt2	N17	89.78(19)
N1	Pt1	O1[1]	90.14(17)	N11[2]	Pt2	N17	90.22(19)
N1[1]	Pt1	N7[1]	89.0(2)	N11[2]	Pt2	O2	91.32(17)
N1	Pt1	N7	89.0(2)	N11	Pt2	O2	88.68(17)
N1	Pt1	N7[1]	91.0(2)	N11	Pt2	O2[2]	91.32(17)
N1[1]	Pt1	N7	91.0(2)	N11[2]	Pt2	O2[2]	88.68(17)
O1	Pt1	O1[1]	180.0	N17[2]	Pt2	N17	180.0
O1	Pt1	N7[1]	89.08(17)	O2[2]	Pt2	N17[2]	89.55(17)
O1	Pt1	N7	90.92(17)	O2	Pt2	N17	89.55(17)
O1[1]	Pt1	N7[1]	90.92(17)	O2	Pt2	N17[2]	90.45(17)
O1[1]	Pt1	N7	89.08(17)	O2[2]	Pt2	N17	90.45(17)
N7[1]	Pt1	N7	180.0(2)	O2	Pt2	O2[2]	180.0
C2	N1	Pt1	126.5(4)	C12	N11	Pt2	125.4(4)
C2	N1	C5	105.2(5)	C12	N11	C15	106.2(5)
C5	N1	Pt1	128.3(4)	C15	N11	Pt2	128.2(4)
N3	C2	N1	111.1(5)	N11	C12	N13	110.0(6)
C2	N3	C4	107.7(5)	C12	N13	C14	108.7(6)
C2	N3	C6	125.7(5)	C12	N13	C16	124.8(6)
C4	N3	C6	126.6(6)	C14	N13	C16	126.5(6)
C5	C4	N3	106.9(5)	C15	C14	N13	105.5(5)
C4	C5	N1	109.1(5)	C14	C15	N11	109.5(5)
N8	N7	Pt1	118.8(4)	N18	N17	Pt2	115.6(4)
N9	N8	N7	174.0(7)	N19	N18	N17	175.1(7)

[1]-X,1-Y,2-Z; [2]-X,1-Y,1-Z

TD-DFT calculations

Table S9. Selected bond distances for complex **18**; N_α, N_β and N_γ labels denote the azido nitrogen atoms. Pt-coordinated N = N_α; central azido N = N_β; terminal N = N_γ.

Bond length	Ground-state (Å)	Lowest-lying excited state(Å)
**Pt–N_α**	2.075	2.435
**Pt–N_α**	2.075	2.526
**Pt–OH**	2.054	2.025
**Pt–OH**	2.031	2.010
**Pt–N(Py)**	2.055	2.062
**Pt–N(Py-CH_3)**	2.068	2.063
**N_α–N_β**	1.217	1.200
**N_α–N_β**	1.217	1.192
**N_β–N_γ**	1.147	1.162
**N_β–N_γ**	1.147	1.167

Table S10. Calculated singlet transitions for complex **18**, in H_2O.

	Energy (eV)	Wavelength (nm)	Oscillator Strength	Major contributions
1	2.96	419	0.004	H-1→LUMO (88%)
2	2.97	417	0.0003	HOMO→LUMO (94%)
3	3.03	409	0.0023	H-2→LUMO (88%)
5	4.08	304	0.0094	H-5→LUMO (57%)<br>H-4→LUMO (26%)
8	4.25	291	0.067	H-3→LUMO (17%)<br>H-2→L+3 (56%)
9	4.30	288	0.127	H-3→LUMO (30%)<br>H-2→L+3 (21%)<br>H-1→L+3 (25%)
13	4.51	275	0.0359	H-5→LUMO (32%)<br>H-4→LUMO (54%)
17	4.76	261	0.1032	H-7→LUMO (38%)<br>H-2→L+2 (21%)
22	5.01	248	0.0379	H-9→LUMO (18%)<br>H-8→LUMO (47%)
30	5.39	230	0.0803	H-5→L+2 (15%)<br>H-4→L+1 (16%)<br>H-4→L+2 (17%)<br>H-1→L+5 (17%)

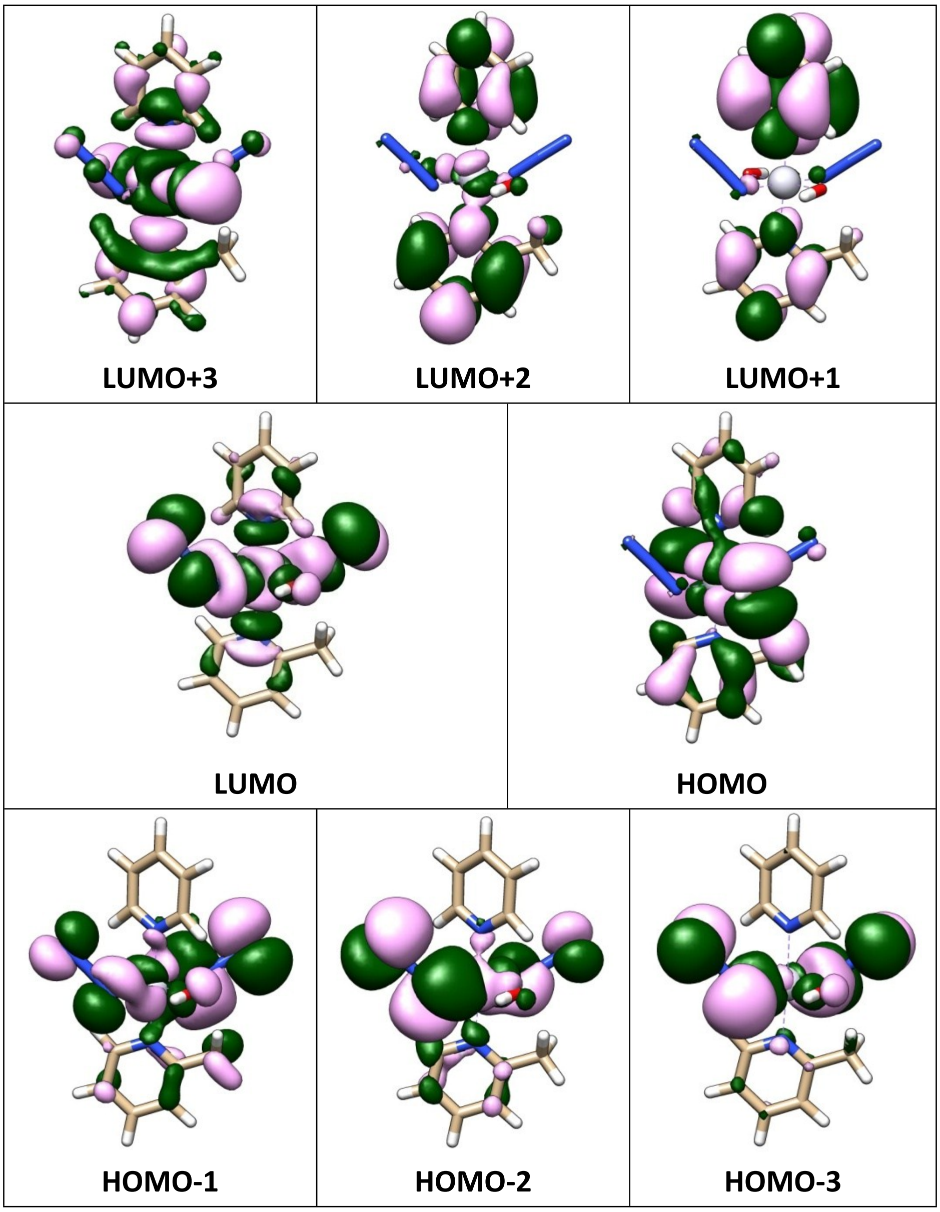

Figure S1: Selected molecular orbitals of complex **18**, with purple and green representing the two signs of the wavefunctions (+ and -).

Irradiation studies with blue light

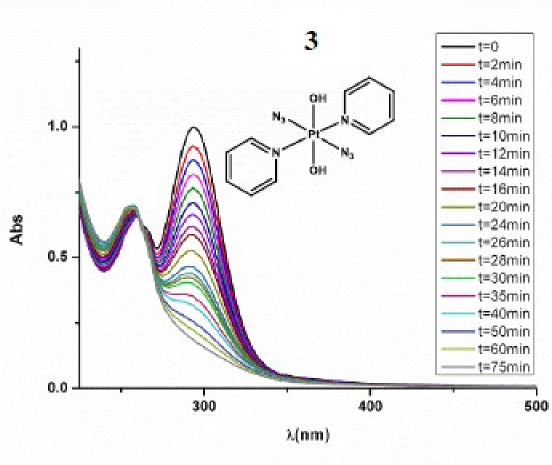

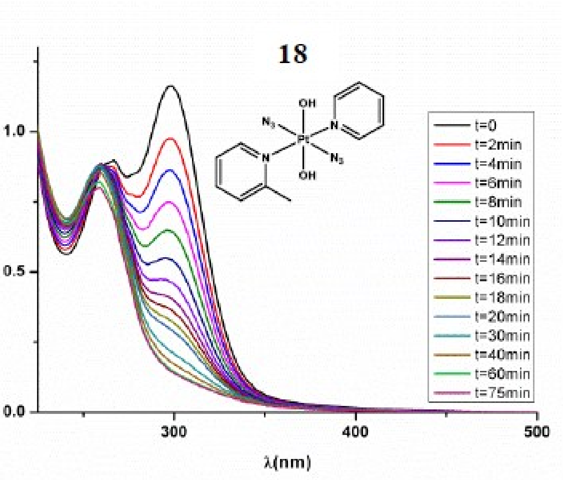

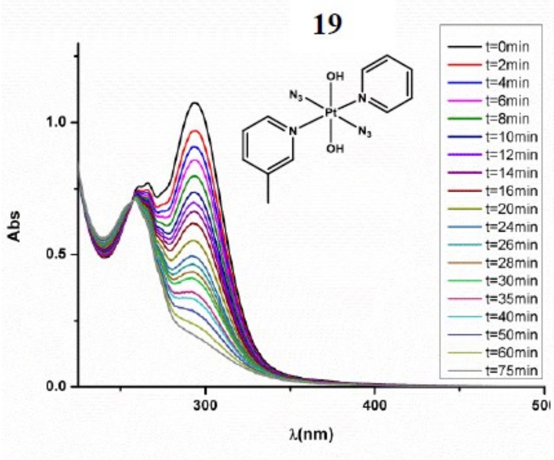

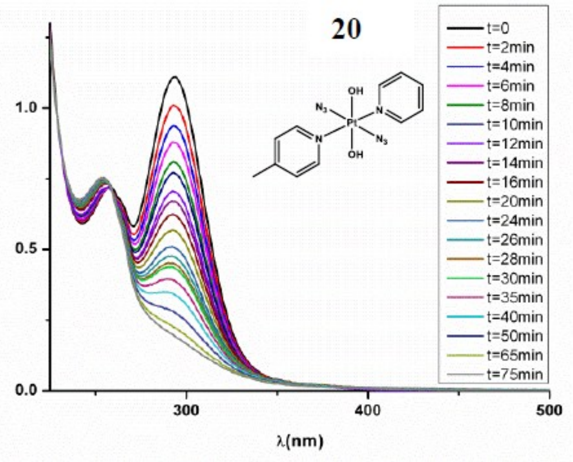

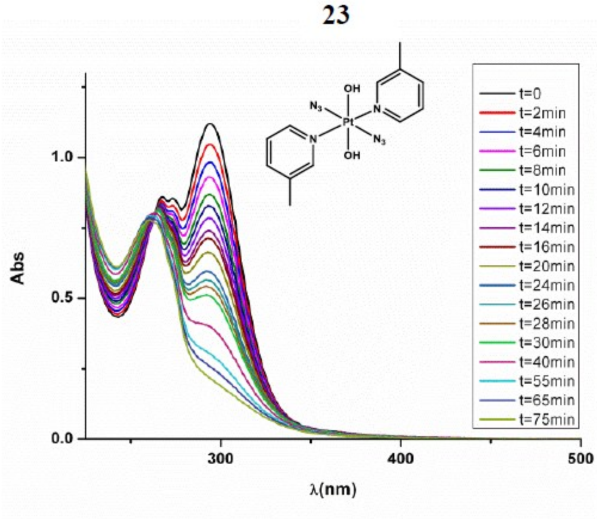

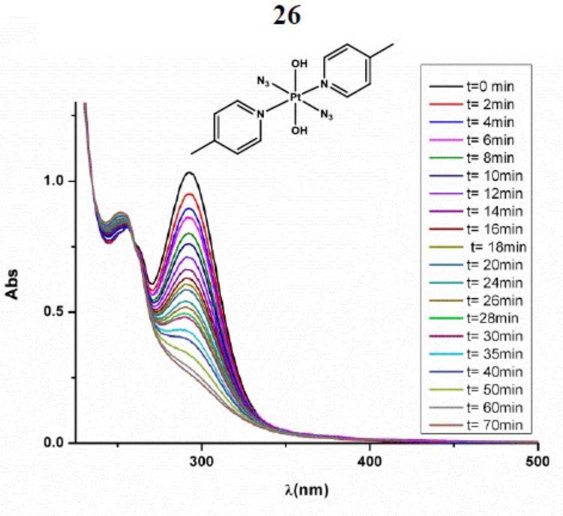

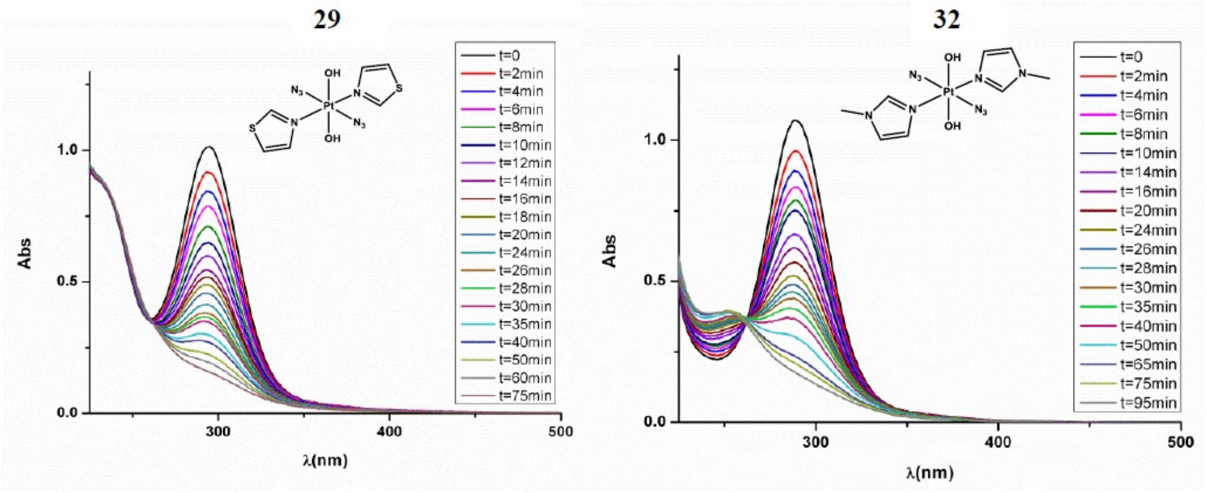

Figure S2: Changes in UV-Vis spectra of the Pt^{IV} diazido complexes (60 μM in H_2O) upon irradiation with 463 nm light.

Complex	$t_{1/2}$ (min)
**18**	9
**19**	21
**20**	20
**23**	26
**26**	25
**29**	17
**32**	22
**3**	22

Table S11. Decomposition half-lives of complexes (60 μM) when irradiated with blue (463 nm) light, as measured by UV-Vis spectroscopy.

Irradiation studies with green light

(a)

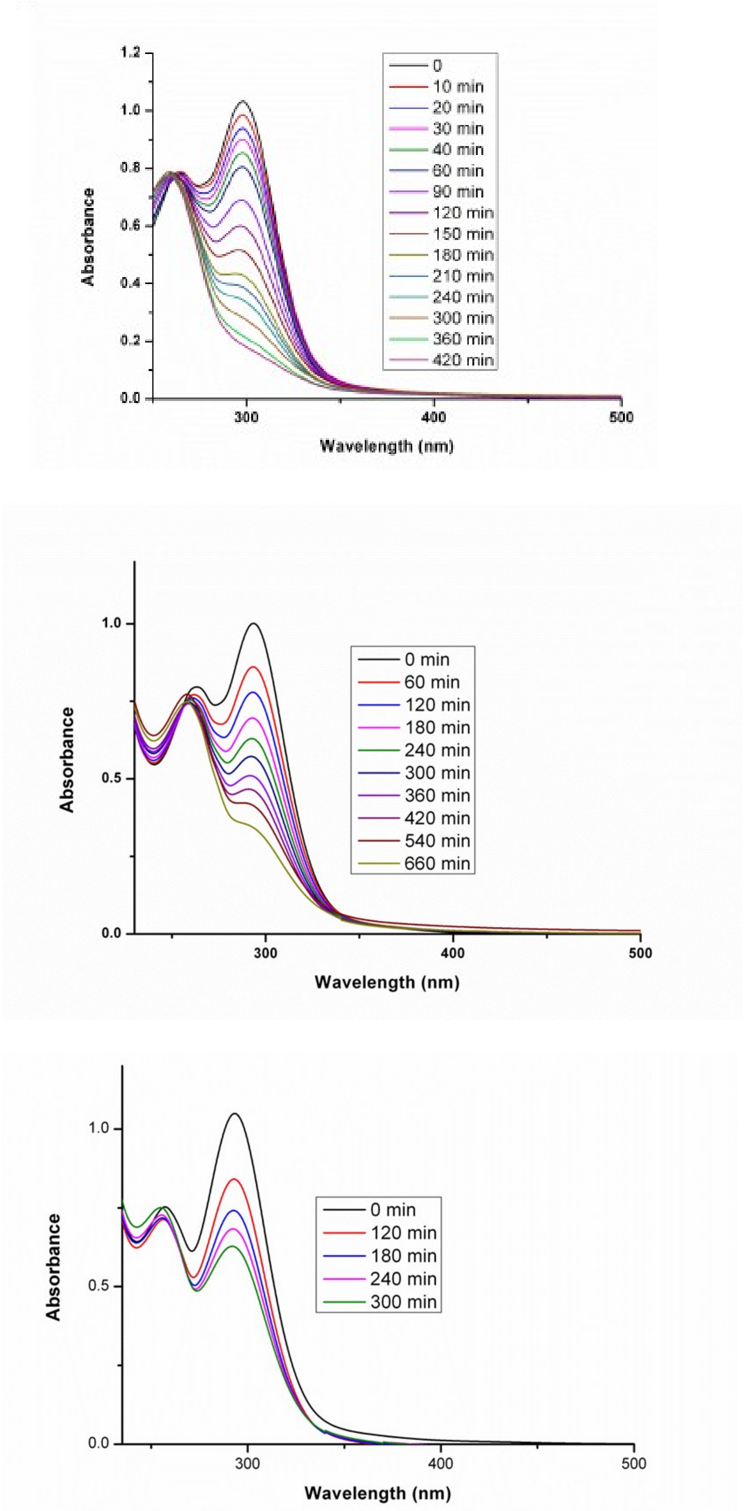

Figure S2: UV-Vis spectra of complexes upon photoirradiation with 517 nm (30 mW.cm^{-2}) showing the decrease in intensity of the N3→Pt LMCT absorption band upon continuous irradiation at *ca.* 20 ^{o}C: (top) *trans, trans, trans*-[Pt(N_3)$_2$(OH)$_2$(py)(2-pic)] **18**, (middle): *trans, trans, trans*-[Pt(N_3)$_2$(OH)$_2$(py)(3-pic)] **19** and (bottom): *trans, trans, trans*-[Pt(N_3)$_2$(OH)$_2$(py)(4-pic)] **20**, respectively.

Complex	%Binding
**18**	52
**19**	58
**20**	64
**23**	68
**26**	64
**29***	80
**32**	48

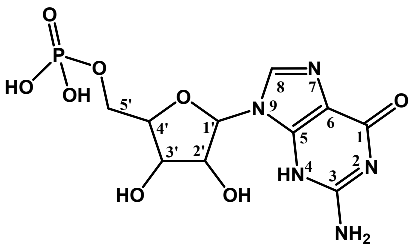

Table S12: 5’-Guanosine monophosphate (5´- GMP) binding of Pt^{IV}-diazido complexes upon blue light irradiation (420 nm, 1 h, 14 $mW.cm^{-2}$) with numbering scheme. The error in % binding is approximated as 5 – 10 % due to the uncertainty in ^{1}H NMR spectral integration of the sugar C_1’ proton.

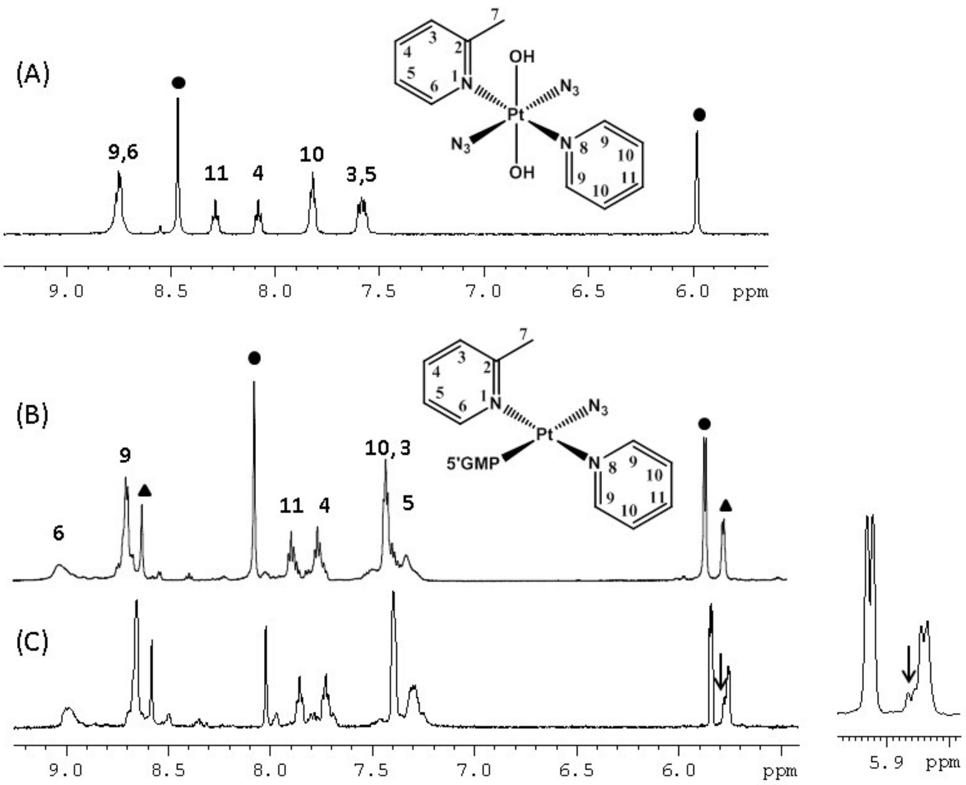

Figure S3: ^{1}H-NMR spectra of complex **18** (9 mM) in the presence of 5'-GMP (2 mol eq) in PBS/D_2O (pH* 7.4); ഊ = free 5'GMP; ① = bound 5'-GMP. (A) Before irradiation. (B) Immediately after irradiation (420 nm, 45 min, 7 mW.cm-^{2}). The formation of the *mono*-5'-GMP adduct is shown, with the assignment of the peaks corresponding to the pyridine ligands on the platinum. Assignment was aided by 2D 1H correlation spectroscopy (COSY). (C) Two weeks after the irradiation, where the evolution of a second (*bis*) 5'-GMP adduct is observed, as shown by the arrows. The spectrum on the right hand side is expanded for the C1 proton region of the sugar ring to illustrate the evolution of the second product 3 days after irradiation.

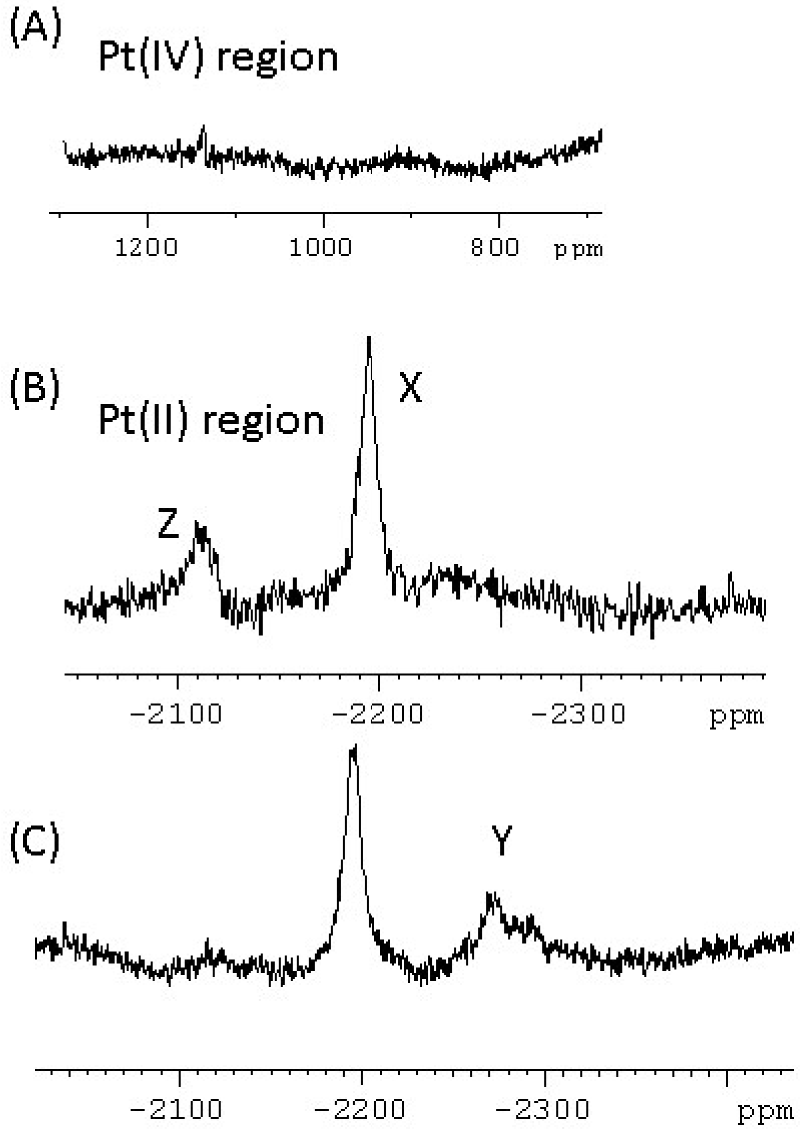

Figure S4: ^{195}Pt NMR spectra of the Pt(IV) and Pt(II) regions after irradiation (420 nm, 45 min, 7 mW.cm^{-2}) of complex **18** (9 mM) in the presence of 5'-GMP. Spectra A and B were obtained immediately after irradiation whereas spectrum C was recorded 2 weeks after the sample was allowed to stand in the dark at ambient temperature. Products **X** and **Y** arw assigned to the *mono*-5'-GMP and *bis*-5'-GMP adducts, respectively. **Z** is tentatively assigned to the intermediate *trans*-[Pt(2-pic)(py)(OH)(5'-GMP).

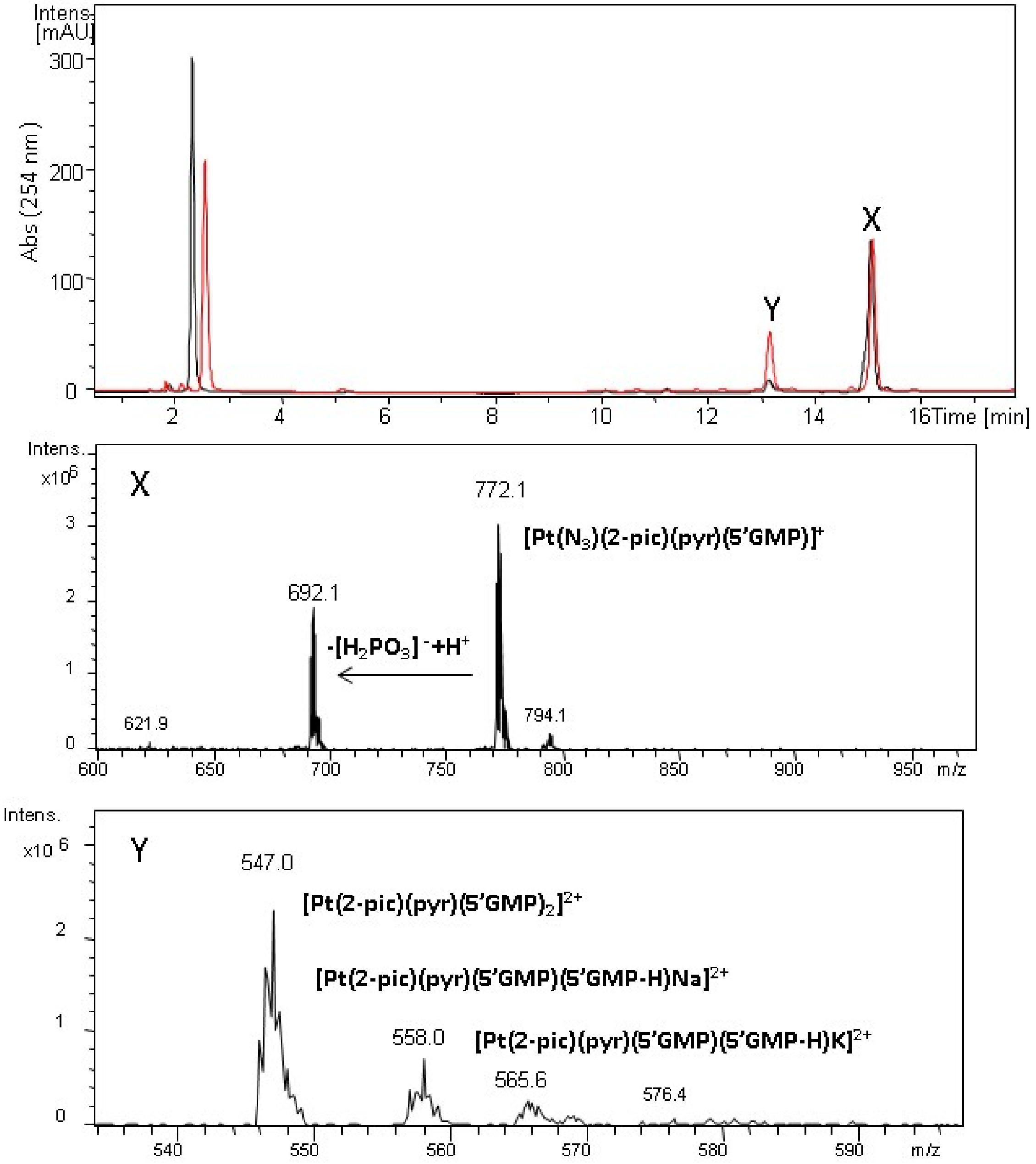

Figure S5: LC-MS of complex **18** (9 mM) irradiated in the presence of 5'-GMP: shortly after irradiation (black) and after two weeks (red). Product **X** (major) corresponds to the *mono*-5'-GMP adduct: $[Pt^{II}(N_3)(py)(2\text{-pic})(5'\text{-GMP})]^+$ (theoretical M= 772.1 *m/z*, $[M\text{-}H_2PO_3^-\text{+}H^+]$= 692.2 *m/z*). Product **Y** evolves over time and is assigned as the *bis*-5'-GMP adduct: $[Pt^{II}(py)(2\text{-pic})(5'\text{-GMP})_2]^{2+}$(theoretical $[M]^{2+}$= 546.6 *m/z*, $[M\text{-}H\text{+}Na]^{2+}$= 557.6 *m/z*, $[M\text{-}H\text{+}K]^{2+}$=565.6 *m/z*).

References

1 N. J. Farrer, J. A. Woods, L. Salassa, Y. Zhao, K. S. Robinson, G. Clarkson, F. S. Mackay and P. J. Sadler, *Angew. Chem. Int. Ed. Engl.*, 2010, **49**, 8905.

2 G. R. Fulmer, A. J. M. Miller, N. H. Sherden, H. E. Gottlieb, A. Nudelman, B. M. Stoltz, J. E. Bercaw and K. I. Goldberg, *Organometallics*, 2010, **29**, 2176.

3 M. J. Frisch, G. W. Trucks and H. B. Schlegel, *Gaussian 03, Revis. D 0.1; Gaussian Inc. Wallingford CT., 2004.*

4 C. Adamo and V. Barone, *J. Chem. Phys.*, 1999, **110**, 6158.

5 P. J. Hay and W. R. Wadt, *J. Chem. Phys.*, 1985, **82**, 270.

6 A. D. McLean and G. S. Chandler, *J. Chem. Phys.*, 1980, **72**, 5639.

7 M. E. Casida, C. Jamorski, K. C. Casida and D. R. Salahub, *J. Chem. Phys.*, 1998, **108**, 4439.

8 R. E. Stratmann, G. E. Scuseria and M. J. Frisch, *J. Chem. Phys.*, 1998, **109**, 8218.

9 O. V. Dolomanov, L. J. Bourhis, R. J. Gildea, J. A. K. Howard and H. Puschmann, *J. Appl. Crystallogr.*, 2009, **42**, 339.

10 G. M. Sheldrick, *Acta Crystallogr. Sect. A Found. Crystallogr.*, 2015, **71**, 3.

11 G. M. Sheldrick, *Acta Crystallogr. Sect. C, Struct. Chem.*, 2015, **71**, 3.

12 H. Moseley, H. Naasan, R. S. Dawe, J. Woods and J. Ferguson, *Photodermatol. Photoimmunol. Photomed.*, 2009, **25**, 8.

13 L. A. Mackenzie and W. Frain-Bell, *Br. J. Dermatol.*, 1973, **89**, 251.

14 F. S. Mackay, J. A. Woods, P. Heringova, J. Kasparkova, A. M. Pizarro, S. A. Moggach, S. Parsons, V. Brabec and P. J. Sadler, *Proc. Natl. Acad. Sci. USA*, 2007, **104**, 20743.

15 G. Repetto, A. del Peso and J. L. Zurita, *Nat. Protoc.*, 2008, **3**, 1125.
